# A Kano model-based demand analysis and perceived barriers of pulmonary rehabilitation interventions for patients with chronic obstructive pulmonary disease in China

**DOI:** 10.1371/journal.pone.0290828

**Published:** 2023-12-18

**Authors:** Xinmeng Yao, Jinmei Li, Jialu He, Qinzhun Zhang, Yi Yu, Yinan He, Jinghua Wu, Weihong Tang, Chengyin Ye

**Affiliations:** 1 Department of Epidemiology and Biostatistics, School of Public Health, Hangzhou Normal University, Hangzhou, Zhejiang, China; 2 Department of Health Management, School of Public Health, Hangzhou Normal University, Hangzhou, Zhejiang, China; 3 Department of Gastroenterology, Hangzhou Children’s Hospital, Hangzhou, Zhejiang, China; University of Chichester - Bishop Otter Campus: University of Chichester, UNITED KINGDOM

## Abstract

**Background:**

Pulmonary rehabilitation (PR) has been recognized to be an effective therapy for chronic obstructive pulmonary disease (COPD). However, in China, the application of PR interventions is still less promoted. Therefore, this cross-sectional study aimed to understand COPD patients’ intention to receive PR, capture the potential personal, social and environmental barriers preventing their willingness of receiving PR, and eventually identify demanding PR services with the highest priority from patients’ point of view.

**Methods:**

In total 237 COPD patients were recruited from 8 health care facilities in Zhejiang, China. A self-designed questionnaire was applied to investigate patients’ intention to participate in PR and potentially associated factors, including personal dimension such as personal awareness, demographic factors, COPD status and health-related literacy/behaviors, as well as social policies and perceived environmental barriers. The demand questionnaire of PR interventions based on the Kano model was further adopted.

**Results:**

Among the 237 COPD patients, 75.1% of COPD patients were willing to participate in PR interventions, while only 62.9% of the investigated patients had heard of PR interventions. Over 90% of patients believed that the cost of PR services and the ratio of medical insurance reimbursement were potential obstacles hindering them from accepting PR services. The multiple linear regression analysis indicated that the PR skills of medical staff, knowledge promotion and public education levels of PR in the community, patients’ transportation concerns and degree of support from family and friends were significantly associated with willingness of participation in PR interventions. By using the Kano model, the top 9 most-requisite PR services (i.e., one-dimensional qualities) were identified from patients’ point of view, which are mainly diet guidance, education interventions, psychological interventions and lower limb exercise interventions. Subgroup analysis also revealed that patients’ demographics, such as breathlessness level, age, education and income levels, could influence their choice of priorities for PR services, especially services related to exercise interventions, respiratory muscle training, oxygen therapy and expectoration.

**Conclusions:**

This study suggested that PR-related knowledge education among patients and their family, as well as providing basic package of PR services with the most-requisite PR items to COPD patients, were considerable approaches to promote PR attendance in the future.

## Introduction

Chronic obstructive pulmonary disease (COPD) is a common respiratory disease that is characterized by persistent airflow limitation, with a progressive decline of lung function [1]. It is recognized as a leading cause of death and disability worldwide, particularly in low- and middle-income countries, inducing a substantial and increasing economic and social burden [2, 3]. In China, the prevalence of COPD among adults over 40 years old has risen by 5.5% in just 10 years (from 8.2% in 2002–2004 to 13.7% in 2012–2015), and is expected to continue to increase due to accelerated population aging in the coming decades [3, 4]. Although pharmacological interventions are widely used in the daily treatment of COPD patients to alleviate symptoms, their impact on improving the long-term declining lung functions remains uncertain [5–7]. On the other hand, comprehensive pulmonary rehabilitation (PR) as a core component of integrated care strategies, has increasingly sparked interest in recent years, and is recommended as a treatment for chronic respiratory disease by American Thoracic Society (ATS) /European Respiratory Society (ERS) [8–10]. PR provides evidence-based therapies that include, but are not limited to, exercise training, education, diet guidance, respiratory training, psychological interventions, oxygen therapy, expectoration guidance and traditional Chinese medicine (TCM)-based non-pharmacological therapy [8, 10–13]. Numerous studies have consistently shown that PR can improve dyspnea, exercise capacity, and overall health status of COPD patients while decreasing healthcare use and associated costs [11, 12, 14, 15].

Currently, successful systematic PR programs have been established in hospitals or community settings in several developed countries [11, 13, 16]. A global survey on content and organization of PR from 40 countries, mainly involving Europe and North America, reported that a median proportion of 75–90% for individuals with chronic respiratory disease successfully completed a PR program [17]. Several key factors, such as perceived benefits of receiving PR and acquired support from family were effective enablers for PR participation [18–20]. On the other hand, the major commonly identified barriers to PR uptake and completion were transport burden, low awareness of PR-related knowledge as well as lack of belief about consequences [18, 19]. The official ATS/ERS statement of 2015 also highlighted important barriers to PR attendance, including insufficient funding, limited resources, insufficient skill of healthcare professionals, and lack of knowledge among patients regarding the benefits of PR [21]. In China, pharmacological treatments are the primary approach to treat stable/acute COPD patients, while the promotion and application of PR interventions are still limited and mostly provided only in hospitals [22]. According to a national epidemiological survey conducted from 2014 to 2015, the rate of respiratory rehabilitation among Chinese COPD patients was found to be 0.8%, and the rate of oxygen inhalation therapy was 5.4% [23]. The study identified regional disparities, lack of COPD knowledge, severe level of mMRC grade and airflow limitation as the primary obstacles to PR utilization. A cross-sectional survey covering 13 hospitals from 2019 to 2020 reported a relatively high PR participation rate of 24.69%, and showed that factors such as awareness level, frequency of hospitalization, and economic status significantly influenced PR attendance [24]. Another cross-sectional study in Xuzhou city in China implied similar findings that patients with heavy economic burden had poor awareness of COPD, resulting in low participation in PR [22]. In summary, these identified influencing factors of PR attendance can be mainly categorized into personal, social and environmental dimensions.

Moreover, only a limited number of demand analyses has been carried out for COPD patients in recent years, which mainly focus on the demand for a singular aspect of PR such as health education on COPD-related information [25–28]. A prospective cohort study explored information needs in COPD patients using the Lung Information Needs Questionnaire (LINQ) and revealed relatively high overall needs for COPD information (mean LINQ overall score: 8.6), with the greatest need being for dietary guidance and self-management [27]. Similarly, a survey of 180 COPD patients admitted to the department of respiratory medicine indicated that over 70% of the patients were eager to receive various health education on reasonable rest, balanced diet, and appropriate exercise [28]. In particular, the most preferred form of health education was a combination of informative manuals provided by hospitals and corresponding explanations provided by medical staff. Currently, there is still a lack of extensive demand research on the various dimensions involved in PR programs. It is crucial for health workers to comprehensively and accurately assess COPD patient needs, identifying individualized requirement items for patients with different characteristics to optimize healthcare resource utilization. Hence, considering challenges such as the lack of research on possible PR interventions in primary healthcare institutions or communities, the contradiction between limited medical resources and complex PR project contents, and the demand to increase the low participation rate of COPD patients in PR in China, this study was introduced from the perspective of COPD patients to both investigate patients’ intentions to participate in PR-related care and identify potential personal, social, and environmental barriers as well as the most required PR services.

Based on this objective, we initially reviewed existing scales related to COPD and found that many well-designed and validated scales primarily focused on assessing COPD knowledge [29–32], quality of life [33–35], self-management [31, 36, 37], and self-efficacy [38, 39] among COPD patients. Specifically, for PR, most scales aimed at evaluating COPD patients’ quality of life and their physiological and psychological changes after receiving PR [39–41]. However, our research specifically aimed to investigate patients’ intention to participate in extensive PR interventions and the potential influencing factors among patients who have not yet received such interventions. Only one study was found addressing a similar purpose, which examined the acceptance of tele-PR by both patients and medical professionals [42], and did not align with our research needs. In short, there is currently no well-established scales that could be directly applied in our study for assessing COPD patients’ intention and various barriers of receiving PR interventions in all aspects. Therefore, integration and optimization of existing questionnaires are required in this study.

The Kano model, proposed by Noriaki Kano, which was a method to identify the attributes of quality, customer needs, and customer satisfaction in quality management, industrial engineering, and business administration, has been expanded to healthcare quality management research [43–51]. Recently, Gómez Martín et al. conducted a patient satisfaction study for the Burn Unit at Getafe University Hospital using the SERVQHOS questionnaire and the Kano questionnaire, ultimately identified the differentiated needs of burn patients and helped improve the hospital’s medical quality [51]. Yuan Yuan et al. designed a Kano need questionnaire of remote nursing services for the elderly in the community, and the results helped medical policy makers and nursing managers to provide targeted nursing services for the elderly [48]. Therefore, the Kano model is recognized as an efficient tool to identify patients’ personalized needs and to prioritize their needs.

In this study, we aimed to investigate the intention of COPD patients to receive PR interventions, analyze the personal, social and environmental factors influencing their intention, most importantly, identify the PR services with the highest priority from patients’ point of view by using the Kano model. It is anticipated that these findings could provide evidence of patients’ most-requisite PR interventions, as well as identify current barriers preventing patients from having PR interventions, eventually giving clues for future PR therapy promotion in community settings in China.

## Materials and methods

### Dataset

**Fig 1:**
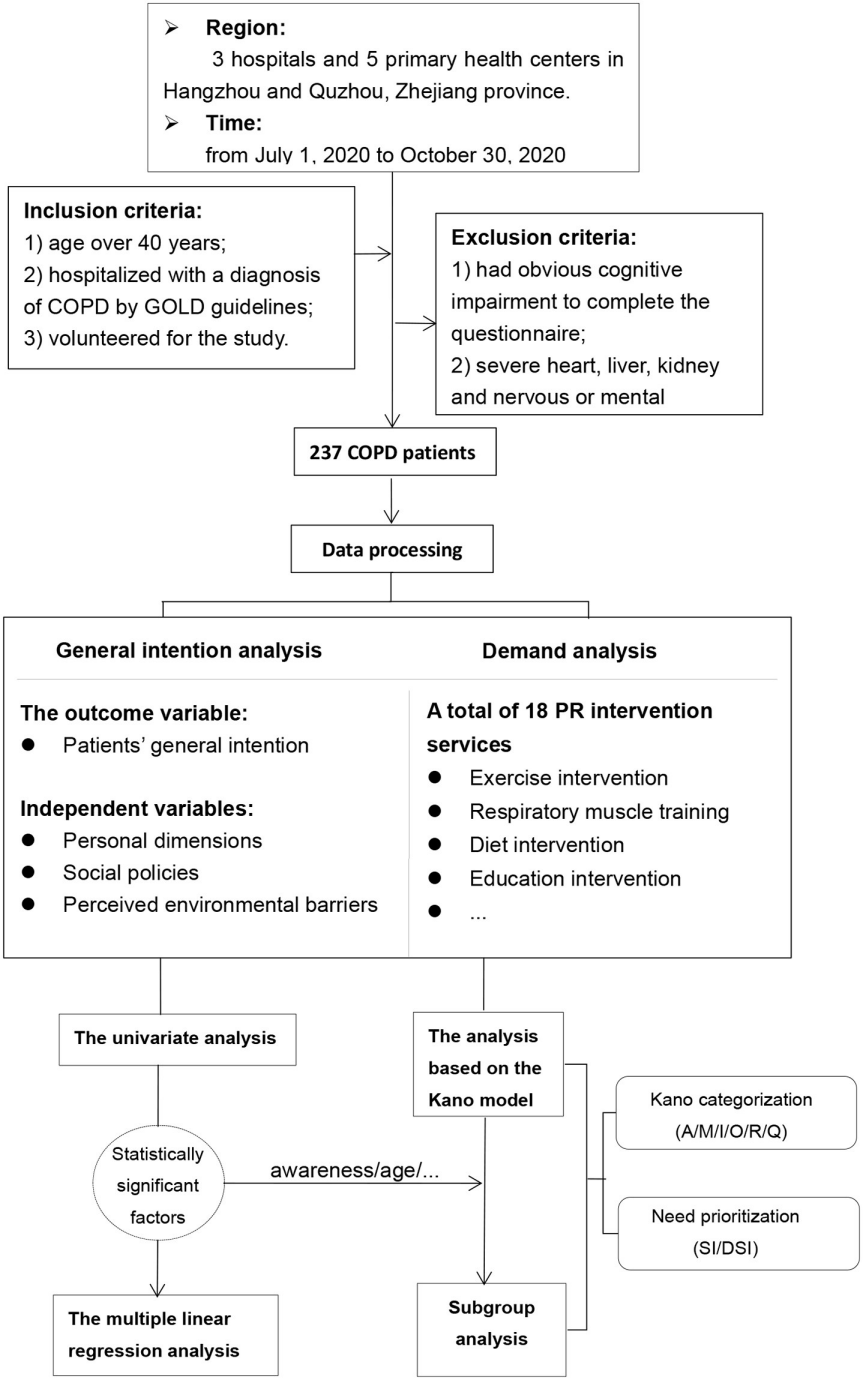
Flow diagram of study.

The study samples were formed by COPD patients that visited 8 health care facilities in cities of Hangzhou and Quzhou, Zhejiang province, including 3 hospitals and 5 primary health centers, from July 1, 2020 to October 30, 2020. Based on the evidence from the 2018 national cross-sectional COPD study in China, it was found that the prevalence of COPD among Chinese adults was significantly higher in individuals over 40 years old (13.7%) compared to those aged 20–39 (2.1%) [4]. In light of this, our study focused primarily on recruiting COPD patients aged 40 years and above to ensure the representativeness of the population, as early PR is recommended for all COPD patients following diagnosis [52, 53]. Detailed inclusion and exclusion criteria were summarized in Fig 1. Moreover, we recognized that the needs and perceived barriers of PR interventions may vary among patients with different ages or COPD severity levels. Therefore, we later conducted series of subgroup analyses to account for these potential differences. A total of 237 COPD patients were investigated by convenience sampling in this study.

The study was conducted according to the guidelines of the Declaration of Helsinki, and was approved by the institutional review board of Hangzhou Normal University (No. 2020115). This study was guided by respiratory physicians and nurses from the investigated health care facilities. All participants were provided with a clear explanation of the study’s objectives and were assured that their data would be kept anonymous, confidential, and solely used for scientific purposes. Before the data collection, verbal informed consent was obtained from each participant.

### Questionnaire design

The questionnaire for this study was designed in two sections. The first section consisted of a self-designed survey on patients’ intention to receive various PR interventions and its potentially relevant factors, covering 3 dimensions: personal, social, and environmental. The personal dimension further included 4 aspects: demographic factors, COPD status, personal awareness of PR, and health-related behaviors. The social dimension mainly involved PR-related social policies such as types of medical insurance enrollment, medical insurance reimbursement and the cost of PR services, while the environmental factors specifically encompassed the PR skills of medical staff, quality of PR provided by medical facilities, knowledge promotion or public education of PR in the community, transportation concerns and degree of support from family and friends. This section integrated several mature scales, such as the modified Medical Research Council dyspnea scale (mMRC) [33] and the social support rating scale (SSRS) [37]. These integrated scales have been all widely used and have demonstrated good reliability and validity among Chinese people. The second section was a demand survey of PR intervention services for COPD patients based on the Kano model. An extensive literature review and relevant guideline reference were conducted [54], and a total of 18 commonly defined PR services were included [8, 10, 11] (S1 File). The reliability of questionnaire was assessed using Cronbach’s α, which yielded a value of 0.800 for the intention and influencing factors questionnaire, 0.831 for functional questions and 0.826 for dysfunctional questions in demand questionnaire. As for validity, the Kaiser-Meyer-Olkin (KMO) measure was employed, yielding values of 0.677, 0.726 and 0.709, respectively. Both the validity and reliability of the questionnaire were acceptable.

### Evaluation strategies according to Kano model

As shown in Table 1, following the steps of the Kano model, 18 attributes of PR services were transferred into a set of functional and dysfunctional questions [54]. Answer options for both types of questions were “Like it”, “Must-be”, “Neutral”, “Accept it” and “Dislike”. There were 5×5 possible results, each of which corresponded to a Kano category [46, 48]. M represented must-be attributes, O represented one-dimensional attributes, A represented attractive attributes, I represented indifferent attributes, R represented reverse attributes and Q represented questionable answers. Subsequently, the frequency of each assigned category was calculated, and the final category was determined to be the one reaching the highest frequency [55]. In detail, the implications of these 6 categories are shown in S2 File. Notably, must-be qualities(M) are the most important qualities and should be provided first, followed by one-dimensional qualities(O) and attractive qualities(A) whereas reverse qualities(R) should be removed from the service list [54, 56].

**Table 1:** Kano evaluation table.

Question type		Dysfunctional: “If (the service) did not satisfy (requirement x), how would you feel?”				
		Like it	Must-be	Neutral	Accept it	Dislike
Functional: “If (the service) satisfied (requirement x), how would you feel?”	Like it	Q	A	A	A	O
	Must-be	R	I	I	I	M
	Neutral	R	I	I	I	M
	Accept it	R	I	I	I	M
	Dislike	R	R	R	R	Q

Q: Questionable, A: Attractive, O: One-dimensional, R: Reverse, I: Indifferent, M: Must-be

The coefficient of patient satisfaction shows how strongly a service influences satisfaction or dissatisfaction, which helps medical staff prioritize patient demands [45]. The coefficient consists of better (satisfaction coefficient, SI) and worse (dissatisfaction coefficient, DSI) values, and the absolute value of the coefficient (ranged from 0 to 1) reflects the level of satisfaction or dissatisfaction with the presence or absence of a service. Specifically, the closer the SI value was to 1, the more satisfied patients were when the target attribute is provided. And the closer the absolute value of DSI was to 1, the more dissatisfied patients were when the target attribute is not provided. If the absolute value is greater than 0.5, an attribute is assumed to be important. The coefficient is calculated as follows [57, 58]:

$$\text{Better}\left(\text{SI}\right)=\;\frac{A+O}{A+O+M+I},\;\text{Worse}\left(\text{DSI}\right)=\frac{O+M}{A+O+M+I}\;\times \left(-1\right)$$

After that, the matrix graph is plotted with worse (DSI) absolute value as x-axis and better (SI) value as y-axis and divided into 4 areas. Concretely, the attributes located in the first quadrant (upper right area), with high absolute values of both SI and DSI, deserve more attention and should be offered with priority to improve satisfaction and reduce dissatisfaction. The attributes located in the second quadrant (upper left area), with higher SI values but lower DSI absolute values, would have a greater impact on satisfaction but less on dissatisfaction. The attributes located in the fourth quadrant (lower right area), with lower SI values but higher DSI absolute values, would have a greater impact on patient dissatisfaction but less on patient satisfaction. The attributes located in the third quadrant (lower left area), with lower absolute values of both SI and DSI, show that they are not important and do not need to improve [56].

### Statistics analysis

Epidata was used for survey data entry, check and error correction. All statistical analysis was carried out using SPSS 22.0 and R software v.4.2.0. Firstly, univariant comparison was conducted in 237 COPD patients. Patients’ intention to receive PR services was treated as the outcome variable, and 23 factors were used as independent variables (S3 File). Quantitative variables were showed as mean ± sd, and categorical variables were showed as n (%). The differences among groups were analyzed by ANOVA (for quantitative variables) and the chi-square/fisher test (for categorical variables). After that, statistically significant variables in the univariant analysis were delivered for the following multiple linear regression analysis, where the patients’ intention was treated as a continuous variable (i.e., coded as 1, 2, 3, 4, and 5 for very low, low, moderate, high, and very high intention, respectively). *P* < 0.05 was considered statistically significant.

## Results

### Subjects’ characteristics and their awareness of PR interventions

Among 237 COPD patients, 156 were residents of Hangzhou, and 81 resided in Quzhou. The male-to-female proportion was 78.5% and 21.5%, respectively. Regarding age distribution, the participants’ ages ranged from 45 to 97, with the majority being over 60 years old (92.5%). The majority (78.5%) achieved educational levels of primary school or junior secondary school, and more than half (51.5%) of the patients had monthly household income between 4000 and 6999 RMB. About 60% investigated patients were in the stage of stable COPD. When using mMRC dyspnea scale to measure the severity of breathlessness, 59.5% of the patients had the mMRC grade of 2 (i.e., walks slower than people of the same age on level ground because of dyspnea or has to stop for breath when walking at own pace). More details could be found in S3 File.

**Fig 2:**
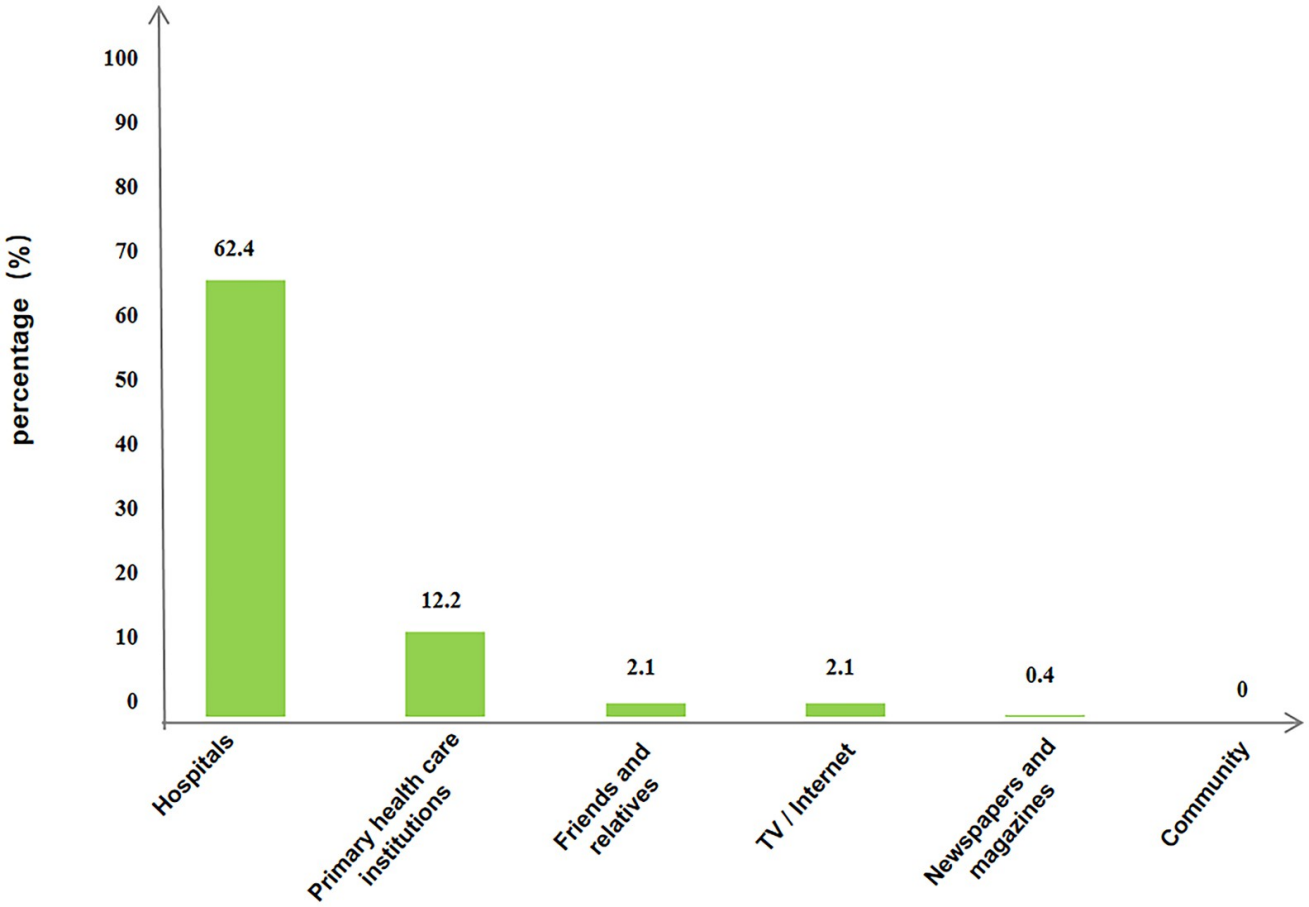
Sources of awareness on PR interventions in COPD patients.

When investigating the overall awareness of PR, 62.9% of the investigated COPD patients were aware of PR interventions (S3 File), mainly informed by medical staff in hospitals (62.4%), followed by primary health centers (12.2%), as shown in Fig 2.

### Analysis of PR intervention intention in COPD patients

When investigating the intention to receive PR interventions, 75.1% of COPD patients reported that they were willing to accept services related to PR interventions. From the patients’ self-reported perspective, it showed that “the cost of PR services” (96.2%), “the ratio of medical insurance reimbursement in PR services” (96.2%), and “the PR skills of medical staff” (95.4%) were recognized as the most important self-reported factors that may influence patients’ intention to accept PR interventions (S3 File).

To comprehensively and objectively explore factors influencing COPD patients’ intention to receive PR services, we also conducted the intention analysis from 3 dimensions: patients’ personal factors, social policies and perceived environmental barriers. The intention to receive PR services was treated as the outcome variable, and factors from the above 3 dimensions were used as independent variables. During the univariate analysis (S3 File), 14 variables were recognized as significant factors associated with the intention, including patients’ personal awareness of the PR interventions, 4 demographic factors (i.e., region, gender, educational level and monthly household income), 4 health-related behaviors (i.e., the drinking habit, emotion management, healthy diet and exercise) and 5 perceived environmental barriers (i.e., PR skills of medical staff, quality of PR services provided by medical facilities, lack of knowledge promotion and public education of PR in the community, transportation concerns and degree of support from family and friends).

Subsequently, the 14 significant factors screened out from the univariate analysis were further introduced into the multiple linear regression analysis. We checked the assumptions of normality, multicollinearity, and autocorrelation before performing multiple linear regression analysis on our data. The residuals were normally distributed, as shown by the Shapiro-Wilk test and S4 File. The Durbin-Watson statistic was 1.824, indicating no autocorrelation. The tolerance values ranged from 0.525 to 0.898, and the VIF values ranged from 1.114 to 1.904, indicating no multicollinearity. These results confirmed that multiple linear regression was appropriate for our data. Ultimately, 6 variables were survived in this step. Specifically, higher PR awareness of patients and better PR skills of medical staff would increase patients’ intention. Patients who had a healthier lifestyle (i.e., no drinking) and received more support from family and friends would have stronger intention. On the contrary, transportation inconvenience and lack of knowledge promotion or public education in the community would reduce the patients’ intention to receive PR interventions (Table 2). Also, multiclass ordinal logistic regression analysis was adopted for comparison. As a result, the significant influencing factors survived in the multiclass ordinal logistic regression analysis were all consistent with those of the multiple linear regression analysis, validating and reinforcing our findings (S5 File).

**Table 2:** Multiple linear regression analysis of COPD patients’ intention to accept PR services.

Variables	Coefficient	Standard error	*t*-value	*P*-value
Constant	2.13	0.76	2.79	0.006
Awareness of PR interventions	0.43	0.10	4.34	<0.001
No drinking	0.14	0.04	3.58	<0.001
The influence of skills of medical staff	0.44	0.09	5.06	<0.001
The influence of knowledge promotion or public education in the community	-0.17	0.04	-3.96	<0.001
The influence of transportation convenience	-0.10	0.04	-2.82	0.005
The influence of support degree from family and friends	0.17	0.05	3.31	<0.001

Considering potential difference in disease progression and symptom severity, we classified patients into two groups: those with less breathlessness (mMRC grade:0–1) and those more breathlessness (mMRC grade:2–4). The intention to receive PR services, as well as personal, social and environmental influencing factors were further examined between the two groups. The results, as shown in S6 File, indicated that there was no significant difference in awareness and intention to receive PR between the two groups. In terms of personal, social and environmental factors, patients with more breathlessness were older, retired, had lower level of education, and showed a higher preference for no smoking. In contrast, patients with less breathlessness placed greater emphasis on maintaining regular exercise and the quality of PR services provided by medical facilities. As our data were collected from two distinct regions of Zhejiang province in China: Hangzhou and Quzhou, we also compared intention to receive PR services, as well as personal, social and environmental influencing factors, between the two regions. The results showed that, a significantly higher proportion of patients living in Hangzhou had stronger intention to receive PR than those living in Quzhou. Notably, there were no significant differences in most patients’ personal influencing factors between the two regions, except for personal awareness. However, there were significant disparities in all of the investigated environmental barriers (S7 File).

### Demand analysis of PR interventions in COPD patients based on Kano model

By using the Kano model, we conducted the demand analysis of PR interventions in COPD patients from 18 attributes, categorized in 8 dimensions (Table 3). As a result, 9 attributes were classified as being of one-dimensional quality, that is, the greater the level to which these PR services were provided, the higher the level of patient satisfaction, and vice versa. These 9 attributes were “provide lower limb interventions”, “inform about prohibited foods”, “education on how to arrange diet properly”, “regularly assess patients’ psychological condition”, “regularly communicate with patients and their family to motivate them for rehabilitation”, “popularize the pathophysiology and clinical basic knowledge of COPD”, “medication instruction”, “personalized guidance on daily life”, and “provide sputum expectoration guidance”. On the other hand, the remaining 9 attributes were classified as being of indifferent quality, in which the quality is not important to COPD patients. These indifferent attributes were services to “provide upper limb exercise interventions”, “provide systemic exercise interventions”, “provide chest breathing training”, “provide pursed lip breathing training”, “provide abdominal breathing training”, “provide long-term oxygen therapy interventions”, “provide non-invasive ventilation interventions”, “dietary guidance according to TCM syndrome differentiation theory”, and “provide TCM non-drug therapy”.

**Table 3:** Evaluation of PR intervention need attributes based on the Kano model.

Dimension	No.	Attributes	A (%)	O (%)	M (%)	I (%)	R (%)	Q (%)	Final category
Exercise	1	Provide upper limb exercise interventions	6 (2.6)	56 (23.6)	1 (0.4)	173 (73)	1 (0.4)	0 (0.0)	I
	2	Provide lower limb exercise interventions	31 (13.1)	143 (60.4)	2 (0.8)	60 (25.3)	1 (0.4)	0 (0.0)	O
	3	Provide systemic exercise interventions	7 (3.0)	85 (35.9)	1 (0.4)	143 (60.3)	1 (0.4)	0 (0.0)	I
Respiratory muscle training	4	Provide chest breathing training	34 (14.4)	97 (40.9)	5 (2.1)	101 (42.6)	0 (0.0)	0 (0.0)	I
	5	Provide pursed lip breathing training	35 (14.8)	97 (40.9)	4 (1.7)	101 (42.6)	0 (0.0)	0 (0.0)	I
	6	Provide abdominal breathing training	35 (14.8)	97 (40.9)	5 (2.1)	100 (42.2)	0 (0.0)	0 (0.0)	I
Diet	7	Inform about prohibited foods	4 (1.7)	204 (86.1)	6 (2.5)	23 (9.7)	0 (0.0)	0 (0.0)	O
	8	Inform about how to properly arrange diet	5 (2.1)	204 (86.1)	6 (2.5)	22 (9.7)	0 (0.0)	0 (0.0)	O
Psychological interventions	9	Regular psychological assessment	21 (8.9)	161 (67.9)	7 (3.0)	47 (19.8)	1 (0.4)	0 (0.0)	O
	10	Regularly communicate with patients and their family to motivate patients for rehabilitation	23 (9.7)	162 (68.4)	7 (3.0)	43 (18.1)	1 (0.4)	1 (0.4)	O
Education	11	Popularize the pathophysiology and clinical basic knowledge of COPD	4 (1.7)	170 (71.7)	39 (16.5)	24 (10.1)	0 (0.0)	0 (0.0)	O
	12	Medication guidance	2 (0.8)	171 (72.2)	40 (16.9)	24 (10.1)	0 (0.0)	0 (0.0)	O
	13	Personalized guidance on daily life	10 (4.2)	183 (77.2)	18 (7.6)	26 (11.0)	0 (0.0)	0 (0.0)	O
Oxygen therapy	14	Provide long-term oxygen therapy interventions	21 (8.9)	88 (37.1)	13 (5.5)	115 (48.5)	0 (0.0)	0 (0.0)	I
	15	Provide non-invasive ventilation interventions	21 (8.9)	88 (37.1)	13 (5.5)	115 (48.5)	0 (0.0)	0 (0.0)	I
Expectoration	16	Provide expectoration guidance	13 (5.5)	126 (53.2)	14 (5.9)	84 (35.4)	0 (0.0)	0 (0.0)	O
TCM-based non-pharmacological	17	Dietary guidance according to TCM syndrome differentiation theory	15 (6.3)	38 (16.1)	4 (1.7)	179 (75.5)	1 (0.4)	0 (0.0)	I
therapy	18	Provide TCM non-drug therapy	15 (6.3)	38 (16.1)	3 (1.3)	180 (75.9)	1 (0.4)	0 (0.0)	I

**Fig 3:**
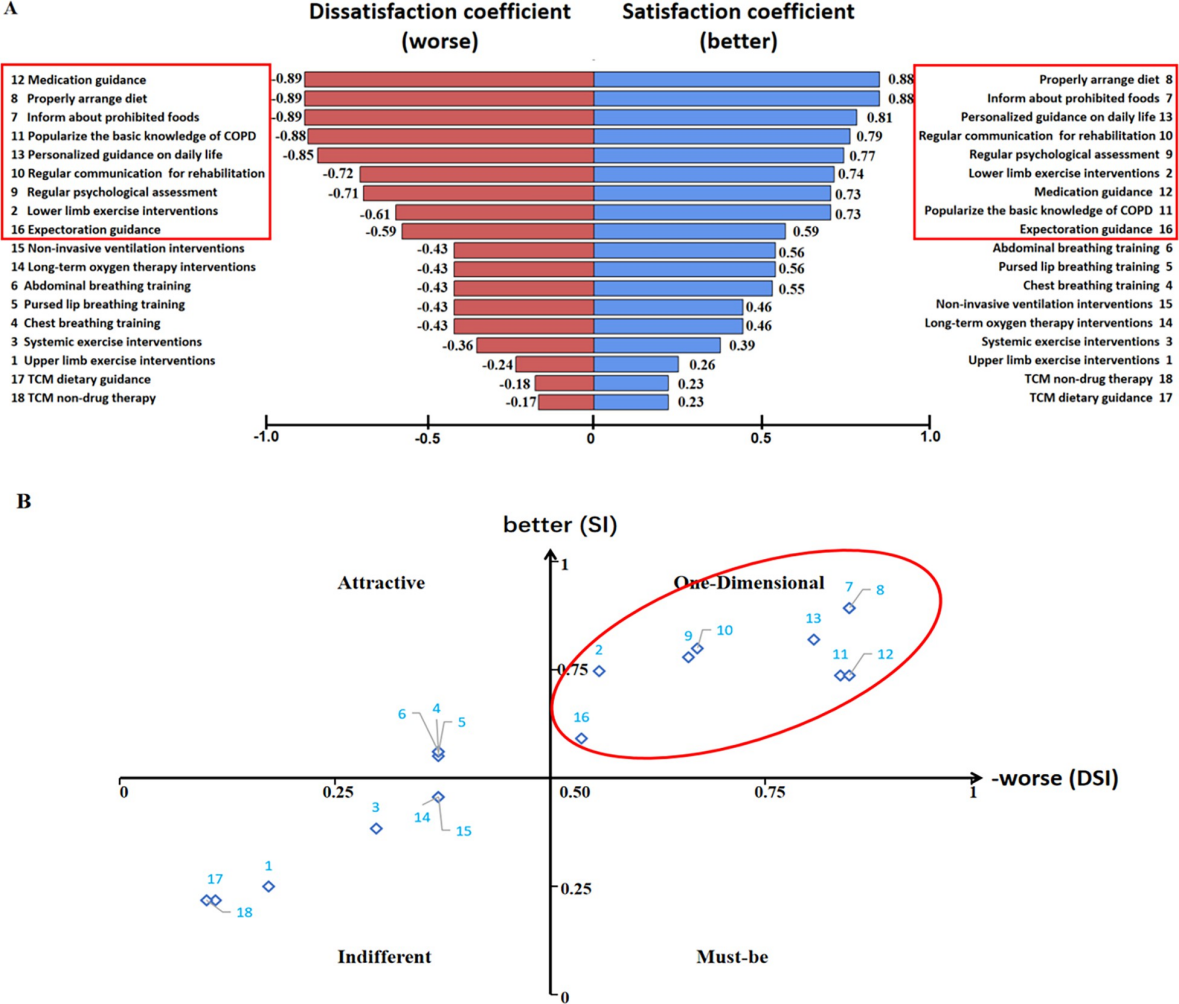
The SI and DSI (A) and their matrix graph (B) of PR needs of COPD patients.

To further clarify which attributes should be provided with the highest priority, the Kano analysis also provided an overview of the degree of satisfaction or dissatisfaction among COPD patients when a certain service was or was not offered (Fig 3). As shown in Fig 3A, the same 9 attributes which were recognized as being of one-dimensional quality above also attained the higher SI, ranged between 0.73 and 0.88, indicating a high degree of satisfaction if they were offered. The same 9 attributes also displayed relatively high absolute value of DSI (all above 0.50), indicating a high level of dissatisfaction if these services were not provided. Similarly, these 9 attributes all exactly located in the upper right section of the matrix graph presented in Fig 3B, which further proved that these services should be provided with the highest priority.

### Comparison of the demands of PR services in various subgroups of COPD patients

To compare Kano quality categories between various subgroups, the chi-square test was performed (Table 4). First, COPD patients were divided into two subgroups according to their awareness of PR (yes or no), and the findings revealed that 6 attributes were assigned to distinct demand categories (“O” and “I”) under these two subgroups, suggesting that these 6 services were in high demand (i.e., one-dimensional quality) for COPD patients who were aware of PR but resulted in indifferent quality for the rest of patients. The identified 6 distinct PR interventions were lower limb exercise interventions, systemic exercise interventions, chest breathing training, pursed lip breathing training, abdominal breathing training and expectoration guidance. Second, based on patients’ intention to receive PR services, we further classified patients into willingness (with “very high” or “high” level of intention) and unwillingness (with “moderate”, “low” or “very low” level of intention) subgroups. As a result, 5 attributes were of greater demand in the willingness subgroup (categorized as “O”) but were of no demand in the unwillingness subgroup (categorized as “I”). All of the 5 attributes were overlapped with the findings from the awareness-subgroup analysis. The details were summarized in Table 4.

**Table 4:** Demand differences of PR interventions in COPD patients in various subgroups.

No.	Item	Kano	Better	Worse	Kano	Better	Worse	Kano	Better	Worse	χ^2^
	Intention	Willingness (N = 178)			Unwillingness (N = 59)						
2	Provide lower limb exercise interventions	O	0.82	-0.67	I	0.48	-0.43				28.22*
4	Provide chest breathing training	O	0.63	-0.51	I	0.31	-0.20				22.95*
5	Provide pursed lip breathing training	O	0.64	-0.50	I	0.31	-0.20				23.65*
6	Provide abdominal breathing training	O	0.64	-0.51	I	0.31	-0.20				23.39*
16	Provide expectoration guidance	O	0.68	-0.68	I	0.31	-0.32				31.94*
	Awareness	Yes (N = 149)			No (N = 88)						
2	Provide lower limb exercise interventions	O	0.87	-0.74	I	0.51	-0.40				40.28*
3	Provide systemic exercise interventions	O	0.54	-0.50	I	0.13	-0.13				45.76*
4	Provide chest breathing training	O	0.66	-0.54	I	0.36	-0.25				24.83*
5	Provide pursed lip breathing training	O	0.67	-0.54	I	0.36	-0.24				25.35*
6	Provide abdominal breathing training	O	0.67	-0.54	I	0.36	-0.25				25.37*
16	Provide expectoration guidance	O	0.72	-0.71	I	0.35	-0.39				36.01*
	Stage of COPD	mMRC: 0–1 (N = 43)			mMRC: 2–4 (N = 194)						
3	Provide systemic exercise interventions	O	0.60	-0.53	I	0.34	-0.33				12.01*
14	Provide long-term oxygen therapy interventions	I	0.19	-0.07	O	0.52	-0.51				27.90*
15	Provide non-invasive ventilation interventions	I	0.19	-0.07	O	0.52	-0.51				27.90*
16	Provide expectoration guidance	I	0.49	-0.40	O	0.61	-0.63				10.00*
	Age	Age<75(N = 104)			Age≥75 (N = 133)						
14	Provide long-term oxygen therapy interventions	I	0.37	0.33	O	0.53	0.50				9.20*
15	Provide non-invasive ventilation interventions	I	0.37	0.33	O	0.53	0.50				9.20*
16	Provide expectoration guidance	I	0.47	0.44	O	0.68	0.71				17.11*
	Household monthly income (RMB)	≤3999 (N = 48)			4000~6999 (N = 122)			≥7000 (N = 67)			
14	Provide long-term oxygen therapy interventions	I	0.33	-0.29	I	0.43	-0.48	O	0.61	-0.43	22.72*
15	Provide non-invasive ventilation interventions	I	0.33	-0.29	I	0.43	-0.48	O	0.61	-0.43	22.72*
16	Provide expectoration guidance	I	0.42	-0.42	O	0.57	-0.60	O	0.73	-0.70	13.19*
	Educational level	Primary schools and below (N = 92)			Junior secondary school (N = 94)			Senior secondary school and above (N = 51)			
14	Provide long-term oxygen therapy interventions	I	0.36	-0.35	I	0.46	-0.47	O	0.65	-0.49	15.12*
15	Provide non-invasive ventilation interventions	I	0.36	-0.35	I	0.46	-0.47	O	0.65	-0.49	15.12*
16	Provide expectoration guidance	I	0.46	-0.47	O	0.64	-0.65	O	0.73	-0.71	15.08*
	Region	Hangzhou (N = 156)			Quzhou (N = 81)						
16	Provide expectoration guidance	O	0.67	-0.63	I	0.42	-0.52				16.63*

*P<0.05

In addition, we divided the investigated COPD patients into subgroups based on several influencing factors, including breathlessness level, age, household monthly income, educational level and region. The results showed that, patients with less breathlessness had a higher demand for systemic exercise interventions, which were categorized as one-dimensional qualities. This was consistent with the findings from the influencing factors section. On the other hand, patients with more breathlessness had a higher demand for the following 3 services: long-term oxygen therapy interventions, non-invasive ventilation interventions, and expectation guidance, all of which were classified as one-dimensional qualities. Likewise, COPD patients with older age (≥75 years old), higher levels of household monthly income(≥7000RMB) and education (Senior secondary school and above) were also found to have high demand for these 3 PR services. There were no substantial disparities in the demand for most PR services between the two regions, except for expectoration guidance. Patients from Hangzhou manifested a stronger need for this service, which was identified as one-dimensional quality, whereas patients in Quzhou considered it an indifferent quality (Table 4). The SI and DSI results also confirmed these findings (S8 File).

## Discussion

### Summary

In this study, firstly, we revealed a low acceptance of PR among Chinese COPD patients and thoroughly investigated the underlying barriers from personal, social, and environmental perspectives. The recognized barriers included limited awareness of PR, concerns about financial burden, transportation difficulties, and lack of family support. Secondly, we applied the Kano model to COPD patients for the first time and identified the 9 most-requisite PR intervention items, categorized into 4 dimensions: lower limb exercise interventions, diet guidance, psychological interventions and education interventions. Meanwhile, our subgroup analyses highlighted the significant demand for certain PR items, including exercise interventions, respiratory muscle training, oxygen therapy, and expectoration, among patients with higher levels of breathlessness, older age and higher income and education levels, this could provide a theoretical basis for designing and delivering tailored PR interventions for specific patients. Based on the findings of the Kano model and the roles/functions of different medical institutions, we proposed here a differentiated hierarchical PR intervention mode, which clarifies the responsibilities of medical institutions at various levels and enables medical staff to promote tailored PR services, forming a whole-cycle continuous PR intervention for COPD patients at different stages.

### Patients’ intention and perceived influencing factors

In our study, 75.1% of COPD patients were willing to participate in PR interventions while the intention still needs to be improved. A cross-sectional survey in China reported the similar findings and further revealed that Chinese COPD patients were not sufficiently aware of the knowledge and importance in PR [24]. This may explain the low willingness and participation rate of PR interventions in China. Another study also reported that lack of knowledge of PR and its benefits was the most frequently identified barriers for low referral to PR [59]. Likewise, our study showed that only 62.9% of the investigated COPD patients were aware of PR interventions, which were mainly informed by hospital medical staff, instead of workers in community-level health centers.

Additionally, over 90% of patients in our study cited the cost of PR services and the ratio of medical insurance reimbursement as major concerns for their intention to participate in PR programs, which indicated that financial status and out-of-pocket costs of PR were the significant factors influencing patients’ perceptions, acceptances and participation in PR [22, 24, 60]. Meanwhile, as observed in our study, the PR skills of medical staff, knowledge promotion or public education of PR in the community, transportation concerns, and degree of support from family and friends, were significantly associated with patients’ intention to accept PR. Our subgroup analysis of different regions also revealed that, compared to patients in Hangzhou, COPD patients residing in the less economically-developed city of Quzhou had significantly lower levels of awareness and willingness to accept PR. They also perceived more environmental barriers, indicating regional disparities in the accessibility and utilization of PR. In summary, these results confirmed that limited environmental resources such as an inadequate medical insurance system [60, 61], inconvenient transportation [20, 61], lack of knowledge regarding the benefits of PR [24, 62], as well as variations in the availability and quality of PR services [24, 62], were major obstacles for the promotion of PR.

Therefore, addressing these environmental barriers should be a priority to increase access to PR for patients living in deprived areas who are at higher risk for hospital admission and poorer health outcomes [63]. For instance, some participants who did not complete the PR program expressed a preference for home-based programs due to transportation burdens and a greater sense of security at home [20]. It was also found that patients and family members who received a family-based PR program had significantly greater improvements in family coping and lower stress levels. This could potentially enhance patients’ beliefs about the consequences and expectations of rehabilitation outcomes and promoting attendance in PR [18, 64]. On the other hand, evidence from other studies suggested that PR could reduce subsequent health resource utilization, such as days spent in the hospital [65, 66], highlighting the importance for education about the potential benefits of PR for COPD patients [20]. Therefore, further efforts should prioritize knowledge promotion and public education of PR in the community for patients and their family. Simultaneously, community-based and family-based PR programs should also be emphasized to facilitate COPD patients’ perceptions, acceptances and participations in PR.

### Patients’ demand

By using the Kano model, we identified most-requisite 9 PR services (i.e., one-dimensional qualities) from patients’ perspective. These services could be classified into 4 categories: diet guidance, education interventions, psychological interventions and lower limb exercise interventions. Our survey found that over 70% of COPD patients had a high level of understanding of these services (i.e., 82.7% for "diet guidance", 78.5% for "psychological interventions" and 70.9% for "exercise interventions"). Malnutrition is a prevalent condition among COPD patients with an incidence ranging from 25% to 65%, and has been identified as an independent risk factor for poor prognosis in COPD, resulting in various adverse consequences, such as prolonged hospitalization, increased family financial burden as well as heightened anxiety [67–69]. Evidence suggests that diet plays a vital role in obstructive lung diseases, including COPD [70, 71]. For instance, a mediterranean-like diet (characterized by high intake of poultry, eggs, fish, vegetables, legumes, potatoes, dairy desserts, fruits, nuts, and dried fruit) may protect lung function [72], while cured meat and processed red meat intake may increase the risk of COPD readmission [73, 74]. Therefore, how to choose an appropriate diet is a common concern and a crucial aspect of daily life for COPD patients, and this may lead to a high demand for dietary guidance in PR interventions. Consistent with the findings in the influencing factors section, health education and psychological guidance offering to COPD patients, especially to their family, have been further proven vital for raising patients’ awareness of PR and strengthening belief of PR effectiveness to promote patients’ willingness to engage in PR programs [61, 64, 75]. In terms of exercise interventions, a prospective cohort study suggested that physical activity is the strongest predictor of all-cause mortality in COPD patients and showed that active participants have a higher probability of survival in comparison to those who are sedentary or very inactive [76]. Dyspnea due to impaired function of ambulation muscles is one of the common symptoms of COPD, and better lower limb muscle function represents the physiological basis of better exercise training in COPD [8, 77]. Therefore, both the above studies and our findings emphasized the importance of lower extremities training.

Meanwhile, the remaining 9 items related to respiratory muscle training, oxygen therapy, TCM-based non-pharmacological therapy and provide upper limb and systemic exercise interventions, were identified as being of indifferent attributes in our study, implying that patients didn’t want to engaged in these services at this stage. However, such low demand may be attributed to patients’ insufficient knowledge of these services, as our findings showing that less than 30% of investigated patients rated themselves as having high-level of cognition of respiratory muscle training and TCM-based non-pharmacological therapy. Based on this finding, we performed subgroup analysis of awareness and intention. The results further proved that higher awareness and willingness would increase patients’ need for lower limb exercise interventions, respiratory muscle training and expectoration guidance, whereas poor perception resulted in the low attendance rate and poor adherence in PR [24]. Therefore, we anticipated that such low demand could be improved through targeted PR education. In addition, our subgroup analysis revealed that a higher level of breathlessness, older age and higher income and education levels would specifically increase patients’ demand for long-term oxygen therapy interventions, non-invasive ventilation interventions, and expectoration guidance. Previous evidence suggested that oxygen therapy was mainly used for exacerbation COPD patients with severe dyspnea symptoms, mostly among older patients, while some of them may also require ventilatory support if they need immediate admission to the respiratory care or intensive care unit (ICU) [78, 79]. However, in our study, there was no significant difference in awareness and intention to receive the overall PR services among patients with different levels of breathlessness, implying the importance of individualized PR strategies. The 2015 official policy statement of enhancing implementation, use, and delivery of PR issued by ATS/ERS stated that patients entering PR programs exhibit heterogeneity regarding disease state, symptoms, as well as functional limitations, and further emphasized giving weight on patient assessments conducted at the start of PR to properly characterize patients and enable delivery of individualized rehabilitation according to each patient’s needs [21]. Furthermore, it has been noted that highly educated patients might have a better understanding of COPD and PR, promoting higher demand [24], while patients with low income tend to have a poor awareness of disease and be vulnerable to adverse circumstances, resulting in lower demand [22, 24, 63]. Therefore, for patients with a higher level of breathlessness, older age, and lower income and education levels, medical staff should specifically emphasize on the severe outcomes of COPD, including respiratory limitations or acute exacerbation. This can help patients understand the importance of long-term oxygen therapy interventions, non-invasive ventilation interventions, and expectoration guidance, so as to improve their participation in PR [23].

### Promotion strategies for PR management

In this study, we found that the awareness rate of PR interventions among COPD patients is considerably low, with the primary source of PR knowledge acquisition being limited to hospitals. However, it is unrealistic for patients to fully understand the importance of PR interventions, develop the necessary skills for PR, and effectively engage in self-management through brief consultations and interactions with healthcare professionals in hospitals alone. As such, there is an urgent need to provide COPD and PR-related health education or knowledge promotion to patients and their family through community-based programs, to help raise their awareness of COPD treatment strategies, as well as the benefits of PR.

Currently, there is a lack of specific guidelines addressing PR promotion for COPD patients. The "Technical Plan for Graded Diagnosis and Treatment Services for Chronic Obstructive Pulmonary Diseases" issued by the General Office of the National Health and Family Planning Commission primarily focuses on the responsibilities of medical institutions based on the complexity of patients’ conditions, without providing a systematic guide for specific PR projects [80]. As a result, most COPD treatments and interventions are still performed by hospitals in China. However, due to the characteristics of chronic respiratory diseases and the need for long-term and continuous rehabilitation, it is difficult for hospitals that mainly treat severe patients, to manage a large number of stable COPD patients simultaneously. According to previous study and our findings, cost and transportation concerns have been identified as major barriers that prevent individuals from PR participation [20, 61]. A more cost-effective community-based mode may be the solution for PR interventions in the near future. However, primary healthcare institutions currently lack the capabilities to provide comprehensive PR services, which may be a great challenge for both community health centers and their medical staff [21]. We proposed here a differentiated hierarchical PR intervention mode based on our findings of the Kano model and the roles/functions of different medical institutions, aiming to not only alleviate the burden on medical staff in all level medical institutions by clarifying their distinct scope of services, but also help to provide tailored PR service packages to improve patient satisfaction and quality of life. Under this hierarchical mode, hospitals mainly provide PR interventions related to long-term oxygen therapy and non-invasive ventilation for severe or exacerbated COPD patients, while stable patients are referred to primary care institutions for continuous PR services involving personalized COPD education, psychological interventions and dietary guidance. Ultimately, this creates a whole-cycle continuous PR intervention for COPD patients at different stages, integrating the resources of both hospitals and primary medical institutions. Notably, novel techniques, such as telerehabilitation, can be incorporated into the community or home-based PR services in primary healthcare facilities as convenient and cost-effective ways to serve COPD patients [81, 82].

It has been demonstrated elsewhere that not all educational and care management programs are appropriate for all patients [83]. To increase the effectiveness and efficiency of PR programs, we suggested that diet guidance, education interventions, psychological interventions and lower limb exercise interventions should be provided to every COPD patient with high priority. Meanwhile, long-term oxygen therapy interventions, non-invasive ventilation interventions, and expectoration guidance may be more important for patients with severe breathlessness or older age. Notably, along with the changing environment and disease progress, the demand attributes identified by the Kano model exhibit a progressive developmental pattern, changing from indifferent demand to attractive demand, one-dimensional demand, and must-be demand, and have a certain life cycle [84]. Consequently, it is crucial to continuously monitor and adjust the proposed strategies of PR services in a dynamic manner, so as to strengthen COPD patients’ motivation to engage in PR interventions, enhance their satisfaction, and improve their health condition and quality of life. Further studies with larger samples are necessary to validate our findings and optimize the individualized PR plans for different COPD patients.

### Limitations

There are some limitations in this study. Firstly, potential bias may exist due to the small sample size, as well as the convenient sampling of COPD patients in hospitals of Zhejiang province of China. Secondly, we explored intention and demand from the level of patients’ perception, while it is also essential to explore current PR services from medical staff’s perspective, and ultimately match the supply and demand from both sides.

## Conclusion

In conclusion, our study revealed that COPD patients’ intention to participate in PR programs remained low in Zhejiang, China. We identified significant obstacles to PR engagement from personal, social and environment dimensions, including patients’ concern of financial burden, low awareness of PR, transportation difficulties, lack of support from family. This suggested that providing preferred health education to patients and their families was critical to improve their understanding of PR. Furthermore, Kano model was first used for COPD patients and successfully identified 9 most-requisite PR services in general population, as well as 3 important PR services for patients with more breathlessness, older age, higher income and education levels, providing clues for subsequent related research and guidance for optimizing individualized PR service packages. We hope this study will assist medical staff in designing targeted PR services for COPD patients, provide effective solutions for PR promotion, and help further improve PR attendance.

## Supporting information

S1 File List of attributes. (DOCX)

S2 File Definition of Kano categories. (DOCX)

S3 File List of characteristics collected from various sources, and their univariant comparison in different intention level. (DOCX)

S4 File Residual distribution for multiple linear regression. (PDF)

S5 File Multiclass ordinal logistic regression analysis of COPD patients’ intention to accept PR services. (DOCX)

S6 File List of characteristics collected from various sources, and their univariant comparison among COPD patients with varying levels of severity. (DOCX)

S7 File List of characteristics collected from various sources, and their univariant comparison in different region. (DOCX)

S8 File The PR intervention demand matrix graphs of COPD patients under different subgroups. (PDF)

S9 File Dataset. (XLSX)

## References

[1] Bonnevie T, Elkins M. Chronic obstructive pulmonary disease. J Physiother. Australian Physiotherapy Association. 2020; 66(1):3–4. doi: 10.1016/j.jphys.2019.09.001 31757773

[2] Ehteshami-Afshar S, FitzGerald JM, Doyle-Waters MM, Sadatsafavi M. The global economic burden of asthma and chronic obstructive pulmonary disease. Int J Tuberc Lung Dis. 2016; 20(1):11–23. doi: 10.5588/ijtld.15.0472 26688525

[3] Christenson SA, Smith BM, Bafadhel M, Putcha N. Chronic obstructive pulmonary disease. Lancet. 2022; 399(10342):2227–42. doi: 10.1016/S0140-6736(22)00470-6 35533707

[4] Wang C, Xu J, Yang L, Xu Y, Zhang X, Bai C, et al. Prevalence and risk factors of chronic obstructive pulmonary disease in China (the China Pulmonary Health [CPH] study): a national cross-sectional study. Lancet. 2018; 391(10131):1706–17. doi: 10.1016/S0140-6736(18)30841-9 29650248

[5] Burge PS, Calverley MA, Jones PW, Spencer S, Anderson JA, Maslen TK. Papers Randomised, double blind, placebo controlled study of fluticasone propionate in patients with moderate to severe chronic obstructive pulmonary disease: the ISOLDE trial. BMJ. 2000; 320(7245):1297–303. doi: 10.1136/bmj.320.7245.1297 10807619 PMC27372

[6] Vestbo J, Sørensen T, Lange P, Brix A, Tone P, Viskum K. Long-term effect of inhaled budesonide in mild and moderate chronic obstructive pulmonary disease: a randomised controlled trial. Lancet. 1999; 353(9167):1819–23. doi: 10.1016/s0140-6736(98)10019-3 10359405

[7] Pauwels RA, Löfdahl CG, Laitinen LA, Schouten JP, Postma DS, Pride NB, et al. Long-term treatment with inhaled budesonide in persons with mild chronic obstructive pulmonary disease who continue smoking. N Engl J Med. 1999; 340(25):1948–53. doi: 10.1056/NEJM199906243402503 10379018

[8] Aizawa H. SYMPOSIUM I 3. COPD: Non-Pharmacologic Treatment. Intern Med. 2007; 46(2):85–6. doi: 10.2169/internalmedicine.46.1778 17220604

[9] Pleguezuelos E, Gimeno-Santos E, Hernández C, Mata M del C, Palacios L, Piñera P, et al. Recommendations on Non-Pharmacological Treatment in Chronic Obstructive Pulmonary Disease From the Spanish COPD Guidelines (GesEPOC 2017). Arch Bronconeumol (Engl Ed). 2018; 54(11):568–75. doi: 10.1016/j.arbres.2018.06.001 30241689

[10] Spruit MA, Singh SJ, Garvey C, Zu Wallack R, Nici L, Rochester C, et al. An official American thoracic society/European respiratory society statement: Key concepts and advances in pulmonary rehabilitation. Am J Respir Crit Care Med. 2013; 188(8):e13–e64. doi: 10.1164/rccm.201309-1634ST 24127811

[11] Nici L, Donner C, Wouters E, Zuwallack R, Ambrosino N, Bourbeau J, et al. American thoracic society/European respiratory society statement on pulmonary rehabilitation. Am J Respir Crit Care Med. 2006; 173(12):1390–413. doi: 10.1164/rccm.200508-1211ST 16760357

[12] COPD Working Group. Pulmonary Rehabilitation for Patients With Chronic Pulmonary Disease (COPD): An Evidence-Based Analysis. Ont Health Technol Assess Ser. 2012; 12(6):1–75 23074434 PMC3384375

[13] Yohannes AM, Connolly MJ. Pulmonary rehabilitation programmes in the UK: A national representative survey. Clin Rehabil. 2004; 18(4):444–9. doi: 10.1191/0269215504cr736oa 15180129

[14] Ko FWS, Cheung NK, Rainer TH, Lum C, Wong I, Hui DSC. Comprehensive care programme for patients with chronic obstructive pulmonary disease: A randomised controlled trial. Thorax. 2017; 72(2):122–8. doi: 10.1136/thoraxjnl-2016-208396 27471050

[15] Van Wetering CR, Hoogendoorn M, Mol SJM, Rutten-Van Mölken MPMH, Schols AM. Short- and long-term efficacy of a community-based COPD management programme in less advanced COPD: A randomised controlled trial. Thorax. 2010; 65(1):7–13. doi: 10.1136/thx.2009.118620 19703824

[16] Johnston CL, Maxwell LJ, Alison JA. Pulmonary rehabilitation in Australia: A national survey. Physiotherapy. 2011; 97(4): 284–290. doi: 10.1016/j.physio.2010.12.001 22051584

[17] Spruit MA, Pitta F, Garvey C, ZuWallack RL, Roberts CM, Collins EG, et al. Differences in content and organisational aspects of pulmonary rehabilitation programmes. Eur Respir J. 2014; 43(5): 1326–37. doi: 10.1183/09031936.00145613 24337043

[18] Cox NS, Oliveira CC, Lahham A, Holland AE. Pulmonary rehabilitation referral and participation are commonly influenced by environment, knowledge, and beliefs about consequences: a systematic review using the Theoretical Domains Framework. J Physiother. 2017; 63(2): 84–93. doi: 10.1016/j.jphys.2017.02.002 28433238

[19] Keating A, Lee A, Holland AE. What prevents people with chronic obstructive pulmonary disease from attending pulmonary rehabilitation? A systematic review. Chron Respir Dis. 2011; 8(2):89–99. doi: 10.1177/1479972310393756 21596892

[20] Keating A, Lee AL, Holland AE. Lack of perceived benefit and inadequate transport influence uptake and completion of pulmonary rehabilitation in people with chronic obstructive pulmonary disease: a qualitative study. J Physiother. 2011; 57(3):183–90. doi: 10.1016/S1836-9553(11)70040-6 21843834

[21] Rochester CL, Vogiatzis I, Holland AE, Lareau SC, Marciniuk DD, Puhan MA, et al. An official American Thoracic Society/European Respiratory Society policy statement: Enhancing implementation, use, and delivery of pulmonary rehabilitation. Am J Respir Crit Care Med. 2015; 192(11): 1373–86. doi: 10.1164/rccm.201510-1966ST 26623686

[22] Lou P, Zhu Y, Chen P, Zhang P, Yu J, Zhang N, et al. Vulnerability, beliefs, treatments and economic burden of chronic obstructive pulmonary disease in rural areas in China: a cross-sectional study. BMC Public Health. 2012; 12:287. doi: 10.1186/1471-2458-12-287 22521113 PMC3436722

[23] Cong S, Wang N, Fan J, Wang BH, Bao HL, Lyu XL, et al. Analysis on respiratory rehabilitation in patients with chronic obstructive pulmonary disease aged 40 years or older in China, 2014–2015. Zhonghua Liu Xing Bing Xue Za Zhi. 2020; 41(7):1014–20. doi: 10.3760/cma.j.cn112338-20200129-00059 32741163

[24] Xie L, Liu Z, Hao S, Wu Q, Sun L, Luo H, et al. Assessment of knowledge, attitude, and practice towards pulmonary rehabilitation among COPD patients: A multicenter and cross-sectional survey in China. Respir Med. 2020; 174:106198. doi: 10.1016/j.rmed.2020.106198 33120194

[25] Jones RCM, Wang X, Harding S, Bott J, Hyland M. Educational impact of pulmonary rehabilitation: Lung Information Needs Questionnaire. Respir Med. 2008; 102(10): 1439–45. doi: 10.1016/j.rmed.2008.04.015 18676136

[26] Hyland ME, Jones RCM, Hanney KE. The Lung Information Needs Questionnaire: Development, preliminary validation and findings. Respir Med. 2006; 100(10): 1807–16. doi: 10.1016/j.rmed.2006.01.018 16524709

[27] Wouters TJ, van Dam van Isselt EF, Achterberg WP. Information needs of older patients living with chronic obstructive pulmonary disease (COPD) indicated for a specific geriatric rehabilitation programme: a prospective cohort study. Int J Palliat Nurs. 2020; 26(5): 238–45. doi: 10.12968/ijpn.2020.26.5.238 32584692

[28] Lihua C, Xiutao C, Xiaoxia LI. Research of health education and information needs in the rehabilitation of COPD patients. Modern Hospital (Chinese); 2012; 12(10): 146–148. Available: https://kns.cnki.net/kcms/detail/detail.aspx?FileName=XDYU201210066&DbName=CJFQ2012

[29] Scott AS, Baltzan MA, Dajczman E, Wolkove N. Patient knowledge in chronic obstructive pulmonary disease: Back to basics. COPD. 2011; 8(5): 375–9. doi: 10.3109/15412555.2011.605402 21936682

[30] Maples P, Franks A, Ray S, Stevens AB, Wallace LS. Development and validation of a low-literacy Chronic Obstructive Pulmonary Disease knowledge Questionnaire (COPD-Q). Patient Educ Couns. 2010; 81(1): 19–22. doi: 10.1016/j.pec.2009.11.020 20044232

[31] Lenferink A, Effing T, Harvey P, Battersby M, Frith P, Van Beurden W, et al. Construct validity of the Dutch version of the 12-item partners in health scale: Measuring patient self-management behaviour and knowledge in patients with chronic obstructive pulmonary disease. PLoS One. 2016; 11(8):e0161595. doi: 10.1371/journal.pone.0161595 27564410 PMC5001637

[32] Wong CKH, Yu WC. Correlates of disease-specific knowledge in Chinese patients with COPD. Int J Chron Obstruct Pulmon Dis. 2016;11: 2221–7. doi: 10.2147/COPD.S112176 27695309 PMC5028094

[33] Jones PW, Adamek L, Nadeau G, Banik N. Comparisons of health status scores with MRC grades in COPD: Implications for the GOLD 2011 classification. Eur Respir J. 2013; 42(3): 647–54. doi: 10.1183/09031936.00125612 23258783

[34] Guyatt GH, Berman LB, Townsend M, Chambers LW. A measure of quality of life for clinical trials in chronic lung disease. Thorax. 1987; 42(10): 773–8. doi: 10.1136/thx.42.10.773 3321537 PMC460950

[35] Hajiro T, Nishimura K, Jones PW, Tsukino M, Ikeda A, Koyama H, et al. A Novel, Short, and Simple Questionnaire to Measure Health-related Quality of Life in Patients with Chronic Obstructive Pulmonary Disease. Am J Respir Crit Care Med. 1999; 159(6):1874–8. doi: 10.1164/ajrccm.159.6.9807097 10351933

[36] Zhang C, Wang W, Li J, Cai X, Zhang H, Wang H, et al. Development and validation of a COPD self-management scale. Respir Care. 2013; 58(11): 1931–6. doi: 10.4187/respcare.02269 23592786

[37] Lan X, Lu X, Yi B, Chen X, Jin S. Factors associated with self-management behaviors of patients with chronic obstructive pulmonary disease. Jpn J Nurs Sci. 2022; 19(1): e12450. doi: 10.1111/jjns.12450 34398525

[38] Wigal JK, Creer TL, Kotses H. The COPD Self-Efficacy Scale. Chest. 1991; 99(5):1193–6. doi: 10.1378/chest.99.5.1193 2019177

[39] Vincent E, Sewell L, Wagg K, Deacon S, Williams J, Singh S. Measuring a Change in Self-Efficacy Following Pulmonary Rehabilitation: An Evaluation of the PRAISE Tool. Chest. 2011; 140(6): 1534–9. doi: 10.1378/chest.10-2649 21737490

[40] Yohannes AM, Roomi J, Winn S, Hans BN, Connolly MJ. The Manchester Respiratory Activities of Daily Living Questionnaire: Development, Reliability, Validity, and Responsiveness to Pulmonary Rehabilitation. J Am Geriatr Soc. 2000; 48(11):1496–500. doi: 10.1111/jgs.2000.48.11.1496 11083331

[41] Yohannes AM, Dryden S, Hanania NA. The Responsiveness of the Anxiety Inventory for Respiratory Disease Scale Following Pulmonary Rehabilitation. Chest. 2016; 150(1): 188–95. doi: 10.1016/j.chest.2016.02.658 26953219

[42] Almojaibel AA, Munk N, Goodfellow LT, Fisher TF, Miller KK, Comer AR, et al. Development and validation of the tele-pulmonary rehabilitation acceptance scale. Respir Care. 2019; 64(9): 1057–64. doi: 10.4187/respcare.06432 30914488

[43] Lin FH, Tsai SB, Lee YC, Hsiao CF, Zhou J, Wang J, et al. Empirical research on Kano’s model and customer satisfaction. PLoS One. 2017; 12(9):e0183888. doi: 10.1371/journal.pone.0183888 28873418 PMC5584930

[44] Mkpojiogu EOC, Hashim NL. Understanding the relationship between Kano model’s customer satisfaction scores and self-stated requirements importance. Springerplus. 2016;5: 197. doi: 10.1186/s40064-016-1860-y 27026893 PMC4769705

[45] Müller SD, Lauridsen KG, Palic AH, Frederiksen LN, Mathiasen M, Løfgren B. Mobile app support for cardiopulmonary resuscitation: Development and usability study. JMIR Mhealth Uhealth. 2021; 9(1):e16114. doi: 10.2196/16114 33399539 PMC7815448

[46] Barrios-Ipenza F, Calvo-Mora A, Criado-García F, Curioso WH. Quality evaluation of health services using the kano model in two hospitals in peru. Int J Environ Res Public Health. 2021; 18(11): 6159. doi: 10.3390/ijerph18116159 34200305 PMC8201113

[47] Sun X, Jia N, Ge L, Liang J. Demand analysis of telenursing among empty-nest elderly individuals with chronic diseases based on the Kano model. Front Public Health. 2022; 10:990295. doi: 10.3389/fpubh.2022.990295 36249233 PMC9555810

[48] Yuan Y, Liu Y, Gong L, Chen H, Zhang S, Kitayama A, et al. Demand Analysis of Telenursing for Community-Dwelling Empty-Nest Elderly Based on the Kano Model. Telemed J E Health. 2021; 27(4): 414–21. doi: 10.1089/tmj.2020.0037 32486912

[49] Mao J, Xie L, Zhao Q, Xiao M, Tu S, Sun W, et al. Demand analysis of an intelligent medication administration system for older adults with chronic diseases based on the Kano model. Int J Nurs Sci. 2022; 9(1): 63–70. doi: 10.1016/j.ijnss.2021.12.012 35079606 PMC8766777

[50] Gustavsson S, Gremyr I, Kenne Sarenmalm E. Using an adapted approach to the Kano model to identify patient needs from various patient roles. TQM Journal. 2016; 28(1): 151–162. doi: 10.1108/TQM-04-2013-0050

[51] Gómez Martín C, García Morato RA, de los Reyes Cortés N, Fernández-Cañamaque JL, Holguín P. Patient satisfaction in a Spanish burn unit. Burns. 2019; 45(2): 341–347. doi: 10.1016/j.burns.2018.03.015 30527645

[52] Ryrsø CK, Godtfredsen NS, Kofod LM, Lavesen M, Mogensen L, Tobberup R, et al. Lower mortality after early supervised pulmonary rehabilitation following COPD-exacerbations: A systematic review and meta-analysis. BMC Pulm Med; 2018; 18(1):154. doi: 10.1186/s12890-018-0718-1 30219047 PMC6139159

[53] Global initiative for chronic obstructive lung disease global strategy for the diagnosis, management, and prevention of chronic obstructive pulmonary disease (2023 report). 2022. www.goldcopd.org

[54] Kermanshachi S, Nipa TJ, Nadiri H. Service quality assessment and enhancement using Kano model. PLoS One. 2022; 17(2):e0264423. doi: 10.1371/journal.pone.0264423 35213604 PMC8880948

[55] Go M, Kim I. In-flight NCCI management by combining the Kano model with the service blueprint: A comparison of frequent and infrequent flyers. Tour Manag. 2018; 69:471–86. doi: 10.1016/j.tourman.2018.06.034

[56] Yao ML, Chuang MC, Hsu CC. The Kano model analysis of features for mobile security applications. Computers and Security. 2018; 78:336–46. doi: 10.1016/j.cose.2018.07.008

[57] Oh J, Yoon S, Park B. A structural approach to examine the quality attributes of e-shopping malls using the Kano model. Asia Pac J Market Lo. 2012; 24(2): 305–27. doi: 10.1108/13555851211218075

[58] Chang WJ, Chang YH. Patient satisfaction analysis: Identifying key drivers and enhancing service quality of dental care. J Dent Sci. 2013; 8(3): 239–47. doi: 10.1016/j.jds.2012.10.006

[59] Milner SC, Boruff JT, Beaurepaire C, Ahmed S, Janaudis-Ferreira T. Rate of, and barriers and enablers to, pulmonary rehabilitation referral in COPD: A systematic scoping review. Respir Med. 2018; 137:103–14. doi: 10.1016/j.rmed.2018.02.021 29605192

[60] Anita Rajagopal RC. Pulmonary Rehabilitation: Improvement with Movement. Chronic Obstr Pulm Dis. 2016; 3(1): 479–84. doi: 10.15326/jcopdf.3.1.2015.0169 28848870 PMC5559130

[61] Oates GR, Niranjan SJ, Ott C, Scarinci IC, Schumann C, Parekh T, et al. Adherence to Pulmonary Rehabilitation in COPD: A QUALITATIVE EXPLORATION of PATIENT PERSPECTIVES on BARRIERS and FACILITATORS. J Cardiopulm Rehabil Prev. 2019; 39(5):344–9. doi: 10.1097/HCR.0000000000000436 31348127 PMC6715533

[62] Barradell AC, Bourne C, Alkhathlan B, Larkin M, Singh SJ. A qualitative assessment of the pulmonary rehabilitation decision-making needs of patients living with COPD. NPJ Prim Care Respir Med. 2022; 32(1):23. doi: 10.1038/s41533-022-00285-9 35768417 PMC9243001

[63] Steiner MC, Lowe D, Beckford K, Blakey J, Bolton CE, Elkin S, et al. Socioeconomic deprivation and the outcome of pulmonary rehabilitation in England and Wales. Thorax. 2017; 72(6): 530–7. doi: 10.1136/thoraxjnl-2016-209376 28077613 PMC5520271

[64] Marquese A, Jácome C, Cruz J, Gabriel R, Brooks D, Figueiredo D. Family-based psychosocial support and education as part of pulmonary rehabilitation in COPD: A randomized controlled trial. Chest. 2015; 147(3): 662–72. doi: 10.1378/chest.14-1488 25340477

[65] Griyths TL, Phillips CJ, Davies S, Burr ML, Campbell IA. Cost effectiveness of an outpatient multidisciplinary pulmonary rehabilitation programme. Thorax. 2001; 56(10): 779–84. doi: 10.1136/thorax.56.10.779 11562517 PMC1745931

[66] Puhan MA, Gimeno-Santos E, Cates CJ, Troosters T. Pulmonary rehabilitation following exacerbations of chronic obstructive pulmonary disease. Cochrane Database Syst Rev. 2016; 12(12):CD005305. doi: 10.1002/14651858.CD005305.pub4 27930803 PMC6463852

[67] Schols AMWJ, Slangen J, Volovics L, Wouters EFM. Weight Loss Is a Reversible Factor in the Prognosis of Chronic Obstructive Pulmonary Disease. Am J Respir Crit Care Med. 1998; 157(6 Pt 1):1791–7. doi: 10.1164/ajrccm.157.6.9705017 9620907

[68] Landbo C, Prescott E, Lange P, Vestbo J, Almdal TP. Prognostic Value of Nutritional Status in Chronic Obstructive Pulmonary Disease. Am J Respir Crit Care Med. 1999; 160(6):1856–61. doi: 10.1164/ajrccm.160.6.9902115 10588597

[69] Vermeeren MAP, Creutzberg EC, Schols AMWJ, Postma DS, Pieters WR, Roldaan AC, et al. Prevalence of nutritional depletion in a large out-patient population of patients with COPD. Respir Med. 2006; 100(8): 1349–55. doi: 10.1016/j.rmed.2005.11.023 16412624

[70] Scoditti E, Massaro M, Garbarino S, Toraldo DM. Role of diet in chronic obstructive pulmonary disease prevention and treatment. Nutrients. 2019; 11(6):1357. doi: 10.3390/nu11061357 31208151 PMC6627281

[71] Zheng PF, Shu L, Si CJ, Zhang XY, Yu XL, Gao W. Dietary Patterns and Chronic Obstructive Pulmonary Disease: A Meta-analysis. COPD. 2016; 13(4):515–22. doi: 10.3109/15412555.2015.1098606 26678388

[72] Sorli-Aguilar M, Martin-Lujan F, Flores-Mateo G, Arija-Val V, Basora-Gallisa J, Sola-Alberich R, et al. Dietary patterns are associated with lung function among Spanish smokers without respiratory disease. BMC Pulm Med. 2016; 16(1): 162. doi: 10.1186/s12890-016-0326-x 27884188 PMC5123418

[73] de Batlle J, Mendez M, Romieu I, Balcells E, Benet M, Donaire-Gonzalez D, et al. Cured meat consumption increases risk of readmission in COPD patients. Eur Respir J. 2012; 40(3): 555–60. doi: 10.1183/09031936.00116911 22408205

[74] Salari-Moghaddam A, Milajerdi A, Larijani B, Esmaillzadeh A. Processed red meat intake and risk of COPD: A systematic review and dose-response meta-analysis of prospective cohort studies. Clin Nutr. 2019;38(3): 1109–16. doi: 10.1016/j.clnu.2018.05.020 29909249

[75] Cosgrove D, MacMahon J, Bourbeau J, Bradley JM, O’Neill B. Facilitating education in pulmonary rehabilitation using the Living Well with COPD programme for pulmonary rehabilitation: A process evaluation. BMC Pulm Med. 2013; 13:50. doi: 10.1186/1471-2466-13-50 23915179 PMC3751129

[76] Waschki B, Kirsten A, Holz O, Müller KC, Meyer T, Watz H, et al. Physical Activity Is the Strongest Predictor of All-Cause Mortality in Patients With COPD: A Prospective Cohort Study. Chest. 2011; 140(2): 331–42. doi: 10.1378/chest.10-2521 21273294

[77] Debigaré Richard M F, Macklem PT. The major limitation to exercise performance in COPD is lower limb muscle dysfunction. Eur J Appl Physiol. 2008; 105(2):751–3. doi: 10.1152/japplphysiol.90336.2008a 18678623

[78] Chronic obstructive pulmonary disease in over 16s: diagnosis and management. London; 2019. 31211541

[79] Austin MA, Wills KE, Blizzard L, Walters EH, Wood-Baker R. Effect of high flow oxygen on mortality in chronic obstructive pulmonary disease patients in prehospital setting: randomised controlled trial. BMJ. 2010; 341:c5462. doi: 10.1136/bmj.c5462 20959284 PMC2957540

[80] Technical Plan for Graded Diagnosis and Treatment Services for Chronic Obstructive Pulmonary Diseases. 2018 [cited 21 Jul 2023]. http://www.nhc.gov.cn/ewebeditor/uploadfile/2018/05/20180502174201649.pdf

[81] Demeyer H, Louvaris Z, Frei A, Rabinovich RA, de Jong C, Gimeno-Santos E, et al. Physical activity is increased by a 12-week semiautomated telecoaching programme in patients with COPD: A multicentre randomised controlled trial. Thorax. 2017; 72(5): 415–23. doi: 10.1136/thoraxjnl-2016-209026 28137918 PMC5520265

[82] Seidman Z, McNamara R, Wootton S, Leung R, Spencer L, Dale M, et al. People attending pulmonary rehabilitation demonstrate a substantial engagement with technology and willingness to use telerehabilitation: a survey. J Physiother. 2017; 63(3): 175–81. doi: 10.1016/j.jphys.2017.05.010 28652080

[83] Kruis AL, Boland MRS, Assendelft WJJ, Gussekloo J, Tsiachristas A, Stijnen T, et al. Effectiveness of integrated disease management for primary care chronic obstructive pulmonary disease patients: Results of cluster randomised trial. BMJ. 2014; 349:g5392. doi: 10.1136/bmj.g5392 25209620 PMC4160285

[84] Löfgren M, Witell L, Gustafsson A. Theory of attractive quality and life cycles of quality attributes. The TQM Journal. 2011; 23(2): 235–46. doi: 10.1108/17542731111110267

