# Supplementary material for: A Kano model-based demand analysis and perceived barriers of pulmonary rehabilitation interventions for patients with chronic obstructive pulmonary disease in China
Source: PLoS One. 2023 Dec 18;18(12):e0290828. doi: 10.1371/journal.pone.0290828 (PMC10727440; doi:10.1371/journal.pone.0290828)
Supplement: S1 File — (DOCX) [file pone.0290828.s001.docx]

**S1 File. List of attributes.** (DOCX)

| Intervention Dimensions | No. | Attributes |
| --- | --- | --- |
| 1. Exercise interventions | (1) | Provide upper limb exercise interventions |
|  | (2) | Provide lower limb exercise interventions |
|  | (3) | Provide systemic exercise interventions |
| 2. Respiratory muscle training | (4) | Provide chest breathing training |
|  | (5) | Provide pursed lip breathing training |
|  | (6) | Provide abdominal breathing training |
| 3. Diet interventions | (7) | Inform about prohibited foods |
|  | (8) | Inform about how to properly arrange diet |
| 4. Psychology interventions | (9) | Regular psychological assessment |
|  | (10) | Regularly communicate with patients and their family to motivate patients for rehabilitation |
| 5. Education interventions | (11) | Popularize the pathophysiology and clinical basic knowledge of COPD |
|  | (12) | Medication guidance |
|  | (13) | Personalized guidance on daily life |
| 6. Oxygen therapy | (14) | Provide long-term oxygen therapy interventions |
|  | (15) | Provide non-invasive ventilation interventions |
| 7. Expectoration | (16) | Provide expectoration guidance |
| 8. TCM-based non-  pharmacological treatment | (17) | Dietary guidance according to TCM syndrome differentiation theory |
|  | (18) | Provide TCM non-drug therapy |
