# Supplementary material for: A Kano model-based demand analysis and perceived barriers of pulmonary rehabilitation interventions for patients with chronic obstructive pulmonary disease in China
Source: PLoS One. 2023 Dec 18;18(12):e0290828. doi: 10.1371/journal.pone.0290828 (PMC10727440; doi:10.1371/journal.pone.0290828)
Supplement: S2 File — (DOCX) [file pone.0290828.s002.docx]

**S2 File. Definition of Kano categories.** (DOCX)

| Kano categories | Definition |
| --- | --- |
| Must-be qualities(M) | Must-be qualities are the basic qualities. This category does not contribute to increased patient satisfaction, but its absence causes high dissatisfaction. |
| One-dimensional qualities(O) | This category causes satisfaction when it presents and causes dissatisfaction when it absents. |
| Attractive qualities(A) | This category can improve patient satisfaction when it is provided, while does not cause dissatisfaction when it is not provided. |
| Indifferent qualities(I) | This category neither causes satisfaction nor dissatisfaction and patients do not care. |
| Reverse qualities(R) | This category results in dissatisfaction when it presents. |
| Questionable qualities(Q) | The responses are invalid. |
