# Supplementary material for: A Kano model-based demand analysis and perceived barriers of pulmonary rehabilitation interventions for patients with chronic obstructive pulmonary disease in China
Source: PLoS One. 2023 Dec 18;18(12):e0290828. doi: 10.1371/journal.pone.0290828 (PMC10727440; doi:10.1371/journal.pone.0290828)
Supplement: S3 File — (DOCX) [file pone.0290828.s003.docx]

**S3 File. List of characteristics collected from various sources, and their univariant comparison in different intention level.** (DOCX)

| Characteristics | Overall  (N=237) | Intention (N=237) | | | | | χ^2^ | *P*-value |
| --- | --- | --- | --- | --- | --- | --- | --- | --- |
|  |  | Very low  (N=3) | Low  (N=4) | Moderate  (N=52) | High  (N=101) | Very high  (N=77) |  |  |
| **1.Personal dimensions** |  |  |  |  |  |  |  |  |
| a) Demographic factors |  |  |  |  |  |  |  |  |
| Region |  |  |  |  |  |  | -3.23 | 0.001 |
| Hangzhou | 156(65.8) | 0(0.0) | 1(25.0) | 29(55.8) | 70(69.3) | 56(72.7) |  |  |
| Quzhou | 81(34.2) | 3(100.0) | 3(75.0) | 23(44.2) | 31(30.7) | 21(27.3) |  |  |
| Gender |  |  |  |  |  |  | -2.19 | 0.030 |
| Male | 186(78.5) | 2(66.7) | 4(100.0) | 33(63.5) | 82(81.2) | 65(84.4) |  |  |
| Female | 51(21.5) | 1(33.3) | 0(0) | 19(36.5) | 19(18.8) | 12(15.6) |  |  |
| Age (years) |  |  |  |  |  |  | 1.79 | 0.074 |
| 40-49 | 5(2.0) | 0(0) | 1(25.0) | 2(3.8) | 1(1.0) | 1(1.3) |  |  |
| 50-59 | 13(5.5) | 0(0) | 0(0) | 4(7.7) | 7(6.9) | 2(2.6) |  |  |
| 60-69 | 50(21.1) | 2(66.7) | 1(25.0) | 10(19.2) | 23(22.8) | 14(18.2) |  |  |
| 70-79 | 72(30.4) | 0(0) | 1(25.0) | 18(34.6) | 28(27.7) | 25(32.5) |  |  |
| 80-89 | 85(35.9) | 0(0) | 1(25.0) | 17(32.7) | 33(32.7) | 34(44.2) |  |  |
| 90~ | 12(5.1) | 1(33.3) | 0(0) | 1(1.9) | 9(8.9) | 1(1.3) |  |  |
| Educational level |  |  |  |  |  |  | 4.59 | <0.001 |
| Primary schools and below | 92(38.8) | 3(100.0) | 2(50.0) | 30(57.7) | 37(36.7) | 20(26.0) |  |  |
| Junior secondary school or vocational senior secondary school | 94(39.7) | 0(0) | 2(50.0) | 14(26.9) | 50(49.5) | 28(36.4) |  |  |
| Senior secondary school | 25(10.5) | 0(0) | 0(0) | 4(7.7) | 8(7.9) | 13(16.9) |  |  |
| Higher vocational school | 7 (3.0) | 0(0) | 0(0) | 3(5.8) | 0(0) | 4(5.1) |  |  |
| Undergraduate or above | 19(8.0) | 0(0) | 0(0) | 1(1.9) | 6(5.9) | 12(15.6) |  |  |
| Occupation |  |  |  |  |  |  | 0.48 | 0.633 |
| Personnel of government organs / state-owned units | 1 (0.4) | 0(0) | 0(0) | 0(0) | 1(1.0) | 0(0) |  |  |
| Corporate employee | 2 (0.9) | 0(0) | 1(25.0) | 0(0) | 0(0) | 1(1.3) |  |  |
| Individually-owned business | 3 (1.3) | 0(0) | 0(0) | 1(1.9) | 0(0) | 2(2.6) |  |  |
| Farmers | 38(16.1) | 0(0) | 1(25.0) | 15(28.8) | 17(16.8) | 5(6.5) |  |  |
| Retiree | 175(73.9) | 3(100.0) | 2(50.0) | 31(59.7) | 73(72.3) | 66(85.7) |  |  |
| Student | 0(0) | 0(0) | 0(0) | 0(0) | 0(0) | 0(0) |  |  |
| Freelance work | 4 (1.7) | 0(0) | 0(0) | 0(0) | 3(3.0) | 1(1.3) |  |  |
| Worker | 14(6.1) | 0(0) | 0(0) | 5(9.6) | 7(6.9) | 2(2.6) |  |  |
| Marriage status |  |  |  |  |  |  | -0.68 | 0.498 |
| Unmarried | 1(0.4) | 0(0) | 0(0) | 0(0) | 0(0) | 1(1.3) |  |  |
| Married | 226(95.4) | 2(66.7) | 4(100.0) | 50(96.2) | 98(97.0) | 72(93.5) |  |  |
| Divorce | 0(0) | 0(0) | 0(0) | 0(0) | 0(0) | 0(0) |  |  |
| Widowed | 10(4.2) | 1(33.3) | 0(0) | 2(3.8) | 3(3.0) | 4(5.2) |  |  |
| Household monthly income (RMB) |  |  |  |  |  |  | 4.27 | <0.001 |
| <1000 | 1 (0.4) | 0(0) | 0(0) | 0(0) | 0(0) | 1(1.2) |  |  |
| 1000-3999 | 47(19.8) | 1(33.3) | 2(50.0) | 13(25.0) | 24(23.8) | 7(9.1) |  |  |
| 4000-6999 | 122(51.5) | 2(66.7) | 1(25.0) | 29(55.8) | 57(56.4) | 33(42.9) |  |  |
| 6000-8999 | 40(16.9) | 0(0) | 0(0) | 9(17.3) | 13(12.9) | 18(23.4) |  |  |
| ＞9000 | 27(11.4) | 0(0) | 1(25.0) | 1(1.9) | 7(6.9) | 18(23.4) |  |  |
| b) COPD status |  |  |  |  |  |  |  |  |
| Stage of COPD |  |  |  |  |  |  |  |  |
| Acute exacerbation period | 95 (40. 1) | 3(100.0) | 1(25.0) | 18(34.6) | 41(40.6) | 32(41.6) | 0.03 | 0.974 |
| Stable period | 142(59.9) | 0(0) | 3(75.0) | 34(65.4) | 60(59.4) | 45(58.4) |  |  |
| Unclear | 0 (0) | 0(0) | 0(0) | 0(0) | 0(0) | 0(0) |  |  |
| mMRC Dyspnea scale |  |  |  |  |  |  | 0.96 | 0.338 |
| 0 | 9 (3.8) | 1(33.3) | 1(25.0) | 3(5.8) | 3(3.0) | 1(1.3) |  |  |
| 1 | 34(14.3) | 0(0) | 0(0) | 8(15.4) | 12(11.8) | 14(18.2) |  |  |
| 2 | 141(59.5) | 1(33.4) | 2(50.0) | 30(57.7) | 64(63.4) | 44(57.1) |  |  |
| 3 | 39(16.5) | 1(33.3) | 1(25.0) | 6(11.5) | 19(18.8) | 12(15.6) |  |  |
| 4 | 14(5.9) | 0(0) | 0(0) | 5(9.6) | 3(3.0) | 6(7.8) |  |  |
| c) Personal awareness of pulmonary  rehabilitation |  |  |  |  |  |  | 10.19 | <0.001 |
| Yes | 149(62.9) | 0(0) | 0(0) | 11(21.2) | 68(67.3) | 70(90.9) |  |  |
| No | 88(37.1) | 3(100.0) | 4(100.0) | 41(78.8) | 33(32.7) | 7(9.1) |  |  |
| d) Health related behaviors |  |  |  |  |  |  |  |  |
| No smoking |  |  |  |  |  |  | 1.91 | 0.057 |
| Always | 203(85.7) | 3(100.0) | 2(50.0) | 41(78.8) | 88(87.1) | 69(89.6) |  |  |
| Usually | 0(0) | 0(0) | 0(0) | 0(0) | 0(0) | 0(0) |  |  |
| Sometimes | 0(0) | 0(0) | 0(0) | 0(0) | 0(0) | 0(0) |  |  |
| Occasionally | 2 (0.8) | 0(0) | 0(0) | 0(0) | 0(0) | 2(2.6) |  |  |
| Never | 32(13.5) | 0(0) | 2(50.0) | 11(21.2) | 13(12.9) | 6(7.8) |  |  |
| No drinking |  |  |  |  |  |  | 4.70 | <0.001 |
| Always | 215(90.7) | 1(33.3) | 1(25.0) | 44(84.6) | 96(95.0) | 73(94.8) |  |  |
| Usually | 1 (0.4) | 0(0) | 0(0) | 0(0) | 0(0) | 1(1.3) |  |  |
| Sometimes | 0(0) | 0(0) | 0(0) | 0(0) | 0(0) | 0(0) |  |  |
| Occasionally | 2 (0.8) | 0(0) | 2(50.0) | 0(0) | 0(0) | 0(0) |  |  |
| Never | 19 (8.0) | 2(66.7) | 1(25.0) | 8(15.4) | 5(5.0) | 3(3.9) |  |  |
| Emotion management |  |  |  |  |  |  | 4.44 | <0.001 |
| Always | 134(56.5) | 2(66.7) | 3(75.0) | 18(34.6) | 49(48.5) | 62(80.5) |  |  |
| Usually | 51(21.5) | 0(0) | 0(0) | 11(21.2) | 34(33.7) | 6(7.8) |  |  |
| Sometimes | 45(19.0) | 1(33.3) | 1(25.0) | 20(38.5) | 17(16.8) | 6(7.8) |  |  |
| Occasionally | 4 (1.7) | 0(0) | 0(0) | 0(0) | 1(1.0) | 3(3.9) |  |  |
| Never | 3 (1.3) | 0(0) | 0(0) | 3(5.8) | 0(0) | 0(0) |  |  |
| Healthy diet |  |  |  |  |  |  | 2.04 | 0.043 |
| Always | 170(71.7) | 3(100.0) | 3(75.0) | 33(63.5) | 64(63.4) | 67(87.0) |  |  |
| Usually | 25(10.5) | 0(0) | 0(0) | 4(7.7) | 19(18.8) | 2(2.6) |  |  |
| Sometimes | 32(13.5) | 0(0) | 0(0) | 12(23.1) | 15(14.9) | 5(6.5) |  |  |
| Occasionally | 5(2.1) | 0(0) | 1(25.0) | 2(3.8) | 1(1.0) | 1(1.3) |  |  |
| Never | 5(2.1) | 0(0) | 0(0) | 1(1.9) | 2(2.0) | 2(2.6) |  |  |
| Exercise |  |  |  |  |  |  | 4.26 | <0.001 |
| Always | 142(59.9) | 1(33.3) | 3(75.0) | 21(40.2) | 60(59.4) | 57(74.0) |  |  |
| Usually | 18(7.6) | 0(0) | 0(0) | 3(5.8) | 12(11.9) | 3(3.9) |  |  |
| Sometimes | 27(11.4) | 0(0) | 0(0) | 10(19.2) | 10(9.9) | 7(9.1) |  |  |
| Occasionally | 15(6.3) | 0(0) | 0(0) | 2(3.8) | 8(7.9) | 5(6.5) |  |  |
| Never | 35(14.8) | 2(66.7) | 1(25.0) | 16(30.8) | 11(10.9) | 5(6.5) |  |  |
| **2.Social policies** |  |  |  |  |  |  |  |  |
| a) Types of insurance |  |  |  |  |  |  | 1.08 | 0.283 |
| Basic medical insurance for urban residents | 48(20.3) | 2(66.7) | 0(0) | 16(30.8) | 25(24.8) | 5(6.5) |  |  |
| Basic medical insurance for urban workers | 130(54.9) | 1(33.3) | 3(75.0) | 20(38.5) | 59(58.4) | 47(61.0) |  |  |
| Commercial insurance | 1 (0.4) | 0(0) | 0(0) | 1(1.9) | 0(0) | 0(0) |  |  |
| Public funded medical care | 20(8.4) | 0(0) | 0(0) | 1(1.9) | 2(2.0) | 17(22.1) |  |  |
| New rural cooperative medical care | 38(16.0) | 0(0) | 1(25.0) | 14(26.9) | 15(14.8) | 8(10.4) |  |  |
| b) Medical insurance reimbursement ratio |  |  |  |  |  |  | -0.10 | 0.918 |
| Very large | 209(88.2) | 2(66.7) | 4(100.0) | 46(88.5) | 93(92.1) | 64(83.1) |  |  |
| Large | 19(8.0) | 0(0) | 0(0) | 2(3.8) | 7(6.9) | 10(13.0) |  |  |
| Moderate | 8(3.4) | 1(33.3) | 0(0) | 4(7.7) | 1(1.0) | 2(2.6) |  |  |
| Small | 0(0) | 0(0) | 0(0) | 0(0) | 0(0) | 0(0) |  |  |
| Very small | 1(0.4) | 0(0) | 0(0) | 0(0) | 0(0) | 1(1.3) |  |  |
| c) Cost of the interventions |  |  |  |  |  |  | -0.11 | 0.914 |
| Very large | 210(88.6) | 2(66.7) | 4(100.0) | 46(88.5) | 94(93.1) | 64(83.1) |  |  |
| Large | 18(7.6) | 0(0) | 0(0) | 2(3.8) | 6(5.9) | 10(13.0) |  |  |
| Moderate | 8(3.4) | 1(33.3) | 0(0) | 4(7.7) | 1(1.0) | 2(2.6) |  |  |
| Small | 0(0) | 0(0) | 0(0) | 0(0) | 0(0) | 0(0) |  |  |
| Very small | 1(0.4) | 0(0) | 0(0) | 0(0) | 0(0) | 1(1.3) |  |  |
| **3.Perceived environmental barriers** |  |  |  |  |  |  |  |  |
| a) The influence of skills of medical staff |  |  |  |  |  |  | 7.34 | <0.001 |
| Very large | 196(82.7) | 1(33.3) | 1(25.0) | 32(61.5) | 90(89.1) | 72(93.5) |  |  |
| Large | 30(12.7) | 0(0) | 2(50.0) | 12(23.1) | 11(10.9) | 5(6.5) |  |  |
| Moderate | 11(4.6) | 2(66.7) | 1(25.0) | 8(15.4) | 0(0) | 0(0) |  |  |
| Small | 0(0) | 0(0) | 0(0) | 0(0) | 0(0) | 0(0) |  |  |
| Very small | 0(0) | 0(0) | 0(0) | 0(0) | 0(0) | 0(0) |  |  |
| b) The influence of interventions service quality provided by medical facilities |  |  |  |  |  |  | -3.41 | <0.001 |
| Very large | 21(8.9) | 1(33.3) | 1(25.0) | 4(7.7) | 4(4.0) | 11(14.2) |  |  |
| Large | 15(6.3) | 1(33.3) | 0(0) | 5(9.6) | 7(6.9) | 2(2.6) |  |  |
| Moderate | 36(15.2) | 1(33.4) | 2(50.0) | 17(32.7) | 13(12.9) | 3(3.9) |  |  |
| Small | 105(44.3) | 0(0) | 0(0) | 18(34.6) | 58(57.4) | 29(37.7) |  |  |
| Very small | 60(25.3) | 0(0) | 1(25.0) | 8(15.4) | 19(18.8) | 32(41.6) |  |  |
| c) The influence of knowledge promotion or public education in the community |  |  |  |  |  |  | -3.92 | <0.001 |
| Very large | 52(21.9) | 1(33.3) | 1(25.0) | 22(42.3) | 20(19.8) | 8(10.3) |  |  |
| Large | 75(31.6) | 1(33.3) | 0(0) | 10(19.2) | 45(44.5) | 19(24.7) |  |  |
| Moderate | 82(34.6) | 1(33.4) | 2(50.0) | 16(30.8) | 31(30.7) | 32(41.6) |  |  |
| Small | 27(11.5) | 0(0) | 1(25.0) | 3(5.8) | 5(5.0) | 18(23.4) |  |  |
| Very small | 1 (0.4) | 0(0) | 0(0) | 1(1.9) | 0(0) | 0(0) |  |  |
| d) The influence of transportation convenience |  |  |  |  |  |  | -6.48 | <0.001 |
| Very large | 29(12.3) | 2(66.7) | 1(25.0) | 15(28.8) | 6(5.9) | 5(6.5) |  |  |
| Large | 31(13.1) | 0(0) | 0(0) | 9(17.3) | 17(16.8) | 5(6.5) |  |  |
| Moderate | 75(31.6) | 1(33.3) | 2(50.0) | 19(36.6) | 36(35.7) | 17(22.0) |  |  |
| Small | 60(25.3) | 0(0) | 0(0) | 5(9.6) | 31(30.7) | 24(31.2) |  |  |
| Very small | 42(17.7) | 0(0) | 1(25.0) | 4(7.7) | 11(10.9) | 26(33.8) |  |  |
| e) The influence of support degree from family and friends |  |  |  |  |  |  | 6.96 | <0.001 |
| Very large | 154(65.0) | 0(0) | 1(25.0) | 22(42.3) | 69(68.3) | 62(80.5) |  |  |
| Large | 35(14.8) | 0(0) | 0(0) | 9(17.3) | 20(19.8) | 6(7.8) |  |  |
| Moderate | 43(18.1) | 2(66.7) | 1(25.0) | 19(36.5) | 12(11.9) | 9(11.7) |  |  |
| Small | 4 (1.7) | 1(33.3) | 1(25.0) | 2(3.8) | 0(0) | 0(0) |  |  |
| Very small | 1 (0.4) | 0(0) | 1(25.0) | 0(0) | 0(0) | 0(0) |  |  |
