# Supplementary figures and images for: A Kano model-based demand analysis and perceived barriers of pulmonary rehabilitation interventions for patients with chronic obstructive pulmonary disease in China
Source: PLoS One. 2023 Dec 18;18(12):e0290828. doi: 10.1371/journal.pone.0290828 (PMC10727440; doi:10.1371/journal.pone.0290828)

**S4 File. Residual distribution for multiple linear regression. (PDF)**

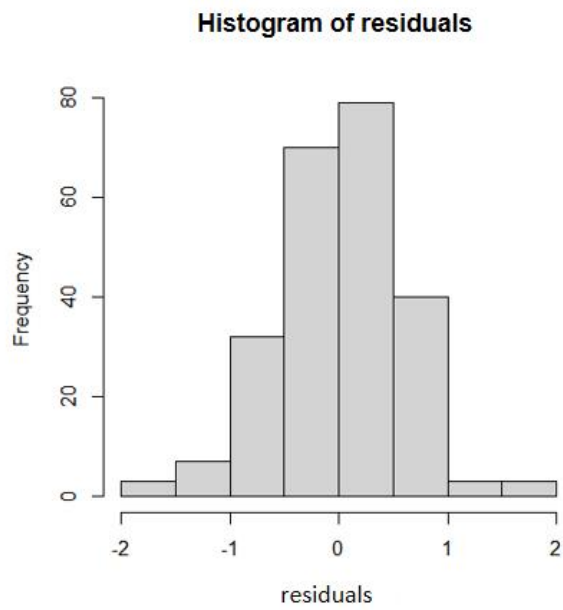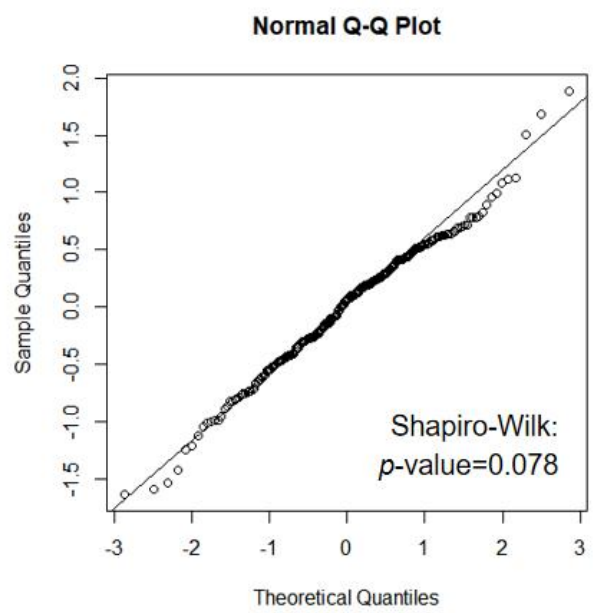

Supplement: S4 File — (PDF) [file pone.0290828.s004.pdf]
