# Supplementary material for: A Kano model-based demand analysis and perceived barriers of pulmonary rehabilitation interventions for patients with chronic obstructive pulmonary disease in China
Source: PLoS One. 2023 Dec 18;18(12):e0290828. doi: 10.1371/journal.pone.0290828 (PMC10727440; doi:10.1371/journal.pone.0290828)
Supplement: S5 File — (DOCX) [file pone.0290828.s005.docx]

**S5 File. Multiclass ordinal logistic regression analysis of COPD patients’ intention to accept PR services.** (DOCX)

| Characteristics | β | OR (95% CI) | *P*-value |
| --- | --- | --- | --- |
| Personal awareness of Pulmonary |  |  |  |
| Yes | 1.79 | 5.96(2.90-12.25) | <0.001 |
| No |  | 1 |  |
| No drinking |  |  |  |
| Yes | -1.90 | 0.15(0.05-0.47) | 0.001 |
| No |  | 1 |  |
| The influence of skills of medical staff ^a^ |  |  |  |
| Large | -3.96 | 0.02(0.01-0.09) | <0.001 |
| Small |  | 1 |  |
| The influence of knowledge promotion or public education in the community |  |  |  |
| Large | 1.29 | 3.64(1.99-6.65) | <0.001 |
| Small |  | 1 |  |
| The influence of transportation convenience |  |  |  |
| Large | 1.08 | 2.93(1.47-5.85) | 0.002 |
| Small |  | 1 |  |
| The influence of support degree from family and friends |  |  |  |
| Large | -1.33 | 0.27(0.12-0.59) | 0.001 |
| Small |  | 1 |  |

^a^ Before the analysis, we recoded some independent variables with multi-levels into binary variables to avoid extremely small counts in certain levels. For instance, we combined “Very large” and “Large” into “Large”, and “Moderate”, “Small”, and “Very small” into “Small” for the question: “does skills of medical staff have an influence on your intention to receive PR interventions?”.
