# Supplementary material for: A Kano model-based demand analysis and perceived barriers of pulmonary rehabilitation interventions for patients with chronic obstructive pulmonary disease in China
Source: PLoS One. 2023 Dec 18;18(12):e0290828. doi: 10.1371/journal.pone.0290828 (PMC10727440; doi:10.1371/journal.pone.0290828)
Supplement: S6 File — (DOCX) [file pone.0290828.s006.docx]

**S6 File. List of characteristics collected from various sources, and their univariant comparison among COPD patients with varying levels of severity.** (DOCX)

| Characteristics | Overall | Level of severity (N=237) | | *t*/χ^2^ | *P*-value |
| --- | --- | --- | --- | --- | --- |
|  |  | mMRC: 0-1  (N=43) | mMRC: 2-4  (N=194) |  |  |
| 1.Intention to receive PR services |  |  |  | 2.73 | 0.553 |
| Very High | 77(32.5) | 15(34.9) | 62(32.0) |  |  |
| High | 101(42.6) | 15(34.9) | 86(44.3) |  |  |
| Moderate | 52(21.9) | 11(25.6) | 41(21.1) |  |  |
| Low | 4(1.7) | 1(2.3) | 3(1.5) |  |  |
| Very Low | 3(1.3) | 1(2.3) | 2(1.0) |  |  |
| 2.Personal dimension |  |  |  |  |  |
| a) Personal awareness of Pulmonary |  |  |  | 0.13 | 0.852 |
| Yes | 149(62.9) | 26 (60.5) | 123(63.4) |  |  |
| No | 88(37.1) | 17 (39.5) | 71(36.6) |  |  |
| b) Demographic factors |  |  |  |  |  |
| Age (years) |  |  |  | 36.99 | <0.001 |
| 40-49 | 5(2.0) | 5 (11.6) | 0 (0.0) |  |  |
| 50-59 | 13(5.5) | 7 (16.3) | 6 (3.1) |  |  |
| 60-69 | 50(21.1) | 13 (30.2) | 37 (19.1) |  |  |
| 70-79 | 72(30.4) | 12 (27.9) | 60 (30.9) |  |  |
| 80-89 | 85(35.9) | 6 (14.0) | 79 (40.7) |  |  |
| 90~ | 12(5.1) | 0 (0.0) | 12 (6.2) |  |  |
| Educational level |  |  |  | 11.56 | 0.021 |
| Primary schools and below | 92(38.8) | 17 (39.5) | 75 (38.7) |  |  |
| Junior secondary school or vocational senior secondary school | 94(39.7) | 19 (44.2) | 75 (38.7) |  |  |
| Senior secondary school | 25(10.5) | 1 (2.3) | 24 (12.4) |  |  |
| Higher vocational school | 7 (3.0) | 4 (9.3) | 3 (1.5) |  |  |
| Undergraduate or above | 19(8.0) | 2 (4.7) | 17 (8.8) |  |  |
| Occupation |  |  |  | 40.37 | <0.001 |
| Personnel of government organs / state-owned units | 1 (0.4) | 0 (0.0) | 1 (0.5) |  |  |
| Corporate employee | 2 (0.9) | 2 (4.7) | 0 (0.0) |  |  |
| Individually-owned business | 3 (1.3) | 0 (0.0) | 3 (1.5) |  |  |
| Farmers | 38(16.1) | 9 (20.9) | 29 (14.9) |  |  |
| Retiree | 175(73.9) | 19 (44.2) | 156 (80.4) |  |  |
| Student | 0(0) | 0(0) | 0(0) |  |  |
| Freelance work | 4 (1.7) | 3 (7.0) | 1 (0.5) |  |  |
| Worker | 14(6.1) | 10 (23.3) | 4 (2.1) |  |  |
| c) Health related behaviors |  |  |  |  |  |
| No smoking |  |  |  | 13.81 | <0.001 |
| Always | 203(85.7) | 29 (67.4) | 174 (89.7) |  |  |
| Usually | 0(0) | 0(0) | 0(0) |  |  |
| Sometimes | 0(0) | 0(0) | 0(0) |  |  |
| Occasionally | 2 (0.8) | 0 (0.0) | 2 (1.0) |  |  |
| Never | 32(13.5) | 14 (32.6) | 18 (9.3) |  |  |
| Exercise |  |  |  | 18.33 | 0.001 |
| Always | 142(59.9) | 38 (88.4) | 104 (53.6) |  |  |
| Usually | 18(7.6) | 2 (4.7) | 16 (8.2) |  |  |
| Sometimes | 27(11.4) | 1 (2.3) | 26 (13.4) |  |  |
| Occasionally | 15(6.3) | 1 (2.3) | 14 (7.2) |  |  |
| Never | 35(14.8) | 1 (2.3) | 34 (17.5) |  |  |
| 3. Social policies |  |  |  |  |  |
| a) Types of insurance |  |  |  | 11.94 | 0.024 |
| Basic medical insurance for urban residents | 48(20.3) | 10 (23.3) | 38 (19.6) |  |  |
| Basic medical insurance for urban workers | 130(54.9) | 21 (48.8) | 109 (56.2) |  |  |
| Commercial insurance | 1 (0.4) | 0 (0.0) | 1 (0.5) |  |  |
| Public funded medical care | 20(8.4) | 0 (0.0) | 20 (10.3) |  |  |
| New rural cooperative medical care | 38(16.0) | 12 (27.9) | 26(13.4) |  |  |
| 4. Perceived environmental barriers |  |  |  |  |  |
| b) The influence of intervention service quality provided by medical facilities |  |  |  | 16.39 | 0.003 |
| Very large | 21(8.9) | 6 (14.0) | 15 (7.7) |  |  |
| Large | 15(6.3) | 8 (18.6) | 7 (3.6) |  |  |
| Moderate | 36(15.2) | 6 (14.0) | 30 (15.5) |  |  |
| Small | 105(44.3) | 16 (37.2) | 89 (45.9) |  |  |
| Very small | 60(25.3) | 7 (16.3) | 53 (27.3) |  |  |
