# Supplementary material for: A Kano model-based demand analysis and perceived barriers of pulmonary rehabilitation interventions for patients with chronic obstructive pulmonary disease in China
Source: PLoS One. 2023 Dec 18;18(12):e0290828. doi: 10.1371/journal.pone.0290828 (PMC10727440; doi:10.1371/journal.pone.0290828)
Supplement: S7 File — (DOCX) [file pone.0290828.s007.docx]

**S7 File.** List of characteristics collected from various sources, and their univariant comparison in different region (There are only important variables with significant differences).

| Characteristics | Overall | Region (N=237) | | *t*/χ^2^ | *P*-value |
| --- | --- | --- | --- | --- | --- |
|  |  | Hangzhou  (N=156) | Quzhou  (N=81) |  |  |
| 1.Intention to receive PR services |  |  |  | 13.25 | 0.01 |
| Very High | 77(32.5) | 56 (35.9) | 21 (25.9) |  |  |
| High | 101(42.6) | 70 (44.9) | 31 (38.3) |  |  |
| Moderate | 52(21.9) | 29 (18.6) | 23 (28.4) |  |  |
| Low | 4(1.7) | 1 (0.6) | 3(3.7) |  |  |
| Very Low | 3(1.3) | 0 (0.0) | 3(3.7) |  |  |
| 2.Personal dimension |  |  |  |  |  |
| a) Personal awareness of pulmonary rehabilitation |  |  |  | 7.91 | 0.005 |
| Yes | 149(62.9) | 108 (69.2) | 41 (50.6) |  |  |
| No | 88(37.1) | 48 (30.8) | 40 (49.4) |  |  |
| 3. Perceived environmental barriers |  |  |  |  |  |
| a) The influence of medical staff skills |  |  |  | 20.08 | <0.001 |
| Very large | 196(82.7) | 139 (89.1) | 57 (70.4) |  |  |
| Large | 30(12.7) | 16 (10.3) | 14 (17.3) |  |  |
| Moderate | 11(4.6) | 1 (0.6) | 10 (12.3) |  |  |
| Small | 0(0) | 0(0) | 0(0) |  |  |
| Very small | 0(0) | 0(0) | 0(0) |  |  |
| b) The influence of intervention service quality provided by medical facilities |  |  |  | 20.07 | <0.001 |
| Very large | 21(8.9) | 10 (6.4) | 11 (13.6) |  |  |
| Large | 15(6.3) | 10 (6.4) | 5 (6.2) |  |  |
| Moderate | 36(15.2) | 20 (12.8) | 16 (19.8) |  |  |
| Small | 105(44.3) | 68 (43.6) | 37 (45.7) |  |  |
| Very small | 60(25.3) | 48 (30.8) | 12 (14.8) |  |  |
| c) The influence of knowledge promotion or public education in the community |  |  |  | 8.33 | 0.016 |
| Very large | 52(21.9) | 35 (22.4) | 17 (21.0) |  |  |
| Large | 75(31.6) | 52 (33.3) | 23 (28.4) |  |  |
| Moderate | 82(34.6) | 51 (32.7) | 31 (38.3) |  |  |
| Small | 27(11.5) | 17 (10.9) | 10 (12.3) |  |  |
| Very small | 1 (0.4) | 1 (0.6) | 0 (0.0) |  |  |
| d) The influence of transportation convenience |  |  |  | 22.78 | <0.001 |
| Very large | 29(12.3) | 8 (5.1) | 21 (25.9) |  |  |
| Large | 31(13.1) | 21 (13.5) | 10 (12.3) |  |  |
| Moderate | 75(31.6) | 51 (32.7) | 24 (29.6) |  |  |
| Small | 60(25.3) | 46 (29.5) | 14 (17.3) |  |  |
| Very small | 42(17.7) | 30 (19.2) | 12 (14.8) |  |  |
| e) The influence of support degree from family and friends |  |  |  | 20.7 | <0.001 |
| Very large | 154(65.0) | 114 (73.1) | 40 (49.4) |  |  |
| Large | 35(14.8) | 22 (14.1) | 13 (16.0) |  |  |
| Moderate | 43(18.1) | 19 (12.2) | 24 (29.6) |  |  |
| Small | 4 (1.7) | 0 (0.0) | 4 (4.9) |  |  |
| Very small | 1 (0.4) | 1 (0.6) | 0 (0.0) |  |  |
